# Comparison between 16S rRNA and shotgun sequencing data for the taxonomic characterization of the gut microbiota

**DOI:** 10.1038/s41598-021-82726-y

**Published:** 2021-02-04

**Authors:** Francesco Durazzi, Claudia Sala, Gastone Castellani, Gerardo Manfreda, Daniel Remondini, Alessandra De Cesare

**Affiliations:** 1 Department of Physics and Astronomy, University of Bologna, 40127 Bologna, Italy; 2 Department of Experimental, Diagnostic and Specialty Medicine, University of Bologna, 40127 Bologna, Italy; 3 Department of Agricultural and Food Sciences, University of Bologna, 40064 Ozzano dell’Emilia, Italy; 4 Department of Veterinary Medical Sciences, University of Bologna, 40064 Ozzano dell’Emilia, Italy

**Keywords:** Next-generation sequencing, Statistics, Microbial ecology

## Abstract

In this paper we compared taxonomic results obtained by metataxonomics (16S rRNA gene sequencing) and metagenomics (whole shotgun metagenomic sequencing) to investigate their reliability for bacteria profiling, studying the chicken gut as a model system. The experimental conditions included two compartments of gastrointestinal tracts and two sampling times. We compared the relative abundance distributions obtained with the two sequencing strategies and then tested their capability to distinguish the experimental conditions. The results showed that 16S rRNA gene sequencing detects only part of the gut microbiota community revealed by shotgun sequencing. Specifically, when a sufficient number of reads is available, Shotgun sequencing has more power to identify less abundant taxa than 16S sequencing. Finally, we showed that the less abundant genera detected only by shotgun sequencing are biologically meaningful, being able to discriminate between the experimental conditions as much as the more abundant genera detected by both sequencing strategies.

## Introduction

The study of the gut bacterial community composition has become a fast-developing field, both for the assessment of possible correlations with human diseases and pathologies^1,2^ and for the examination of the effects of diets and probiotics in animal productions^3,4^. This developing field has been hugely affected by the emergence and optimization of high-throughput sequencing, that made metagenomics the key instrument to access complex ecosystems, such as the human and animal gut. The popularity of high-throughput sequencing is due to decreasing cost and augmented speed and scalability of experiments^5^, which are crucial aspects when researchers design a sequencing project.

The main objectives of gut metagenomic studies are: (i) the identification of the gut microbiota taxonomic composition, (ii) the characterization of the relative abundances of taxa, (iii) the description of the functional contribution of each taxon and (iv) the understanding of the intra-species and/or intra-population gene heterogeneity^6^. To this aim, metataxonomics and metagenomics strategies are used. Metataxonomics consists in the targeted sequencing of 16S rRNA gene hypervariable regions^7^, and allows representative bacterial taxonomic estimation^8^ even when a relatively small number of raw reads is obtained (i.e., as low as 18,000–20,000 reads per sample)^9,10^. The overall sequencing output of metataxonomics is a set of clusters of nearly identical sequences, referred to as Operational Taxonomic Units (OTUs)^11^ or Amplicon Sequence Variants (ASVs)^12^. From the analysis of such clusters, information on the community diversity, richness and evenness can be derived^13^, while accounting for the degree of divergence between different ecosystems or sample types^14^. However, the choice of primers used to amplify 16S rRNA leads to potential biases in the representation of the taxonomic units^15–18^.

Besides the mapping of the taxonomic composition of a sample, the most challenging task for metagenomic studies is the evaluation of the genic contribution of each member of the investigated community in terms of functional genes^6^. To address this issue, shotgun metagenomic sequencing is the most suitable strategy. Here, long DNA molecules, such as complete chromosomes, are randomly broken into fragments that are then sequenced^19^. Hence, metagenomic data deliver knowledge on the taxonomic composition of the ecosystem under study but also on functional genes in the sample, an information that is not retrievable with 16S rRNA gene sequencing. On the other hand, shotgun metagenomics requires higher coverage than metataxonomics^16^.

In a previous study we gained insights into the effects of *Lactobacillus acidophilus* D2/CSL (CECT 4529) (LA) on the ecology of the bacteria colonising the chicken gastrointestinal (GI) tract^20^. Specifically, we investigated the crop and caeca microbiomes in treated animals and in a control group at 1, 14 and 35 days of rearing, using shotgun metagenomic sequencing. In the present study, the same DNA samples investigated in the previous research were analysed using the targeted 16S rRNA gene sequencing (16S). Then, the results obtained with both sequencing strategies were compared to answer three broad questions: (1) what is the resolution of bacterial populations observed by shotgun sequencing as a function of the total number of reads; (2) how many bacterial genera are retrieved exclusively by one sequencing strategy and not by the other; (3) how much the two sequencing strategies retrieve information about the specific experimental conditions, namely the different compartments of gastrointestinal tracts and the sampling time. To address these questions, we studied the dependence between the capability of detecting bacterial populations and the total number of reads and we showed that, when a sufficient number of reads is available, shotgun sequencing finds a statistically significant higher number of taxa than 16S sequencing, corresponding to the less abundant. Finally, we analysed bacterial community profiles exclusive to each strategy, demonstrating that the genera detected only by shotgun sequencing are able to discriminate between the experimental conditions better than those detected only by 16S sequencing.

## Results and discussion

### Relative species abundance distribution

**Figure 1:**
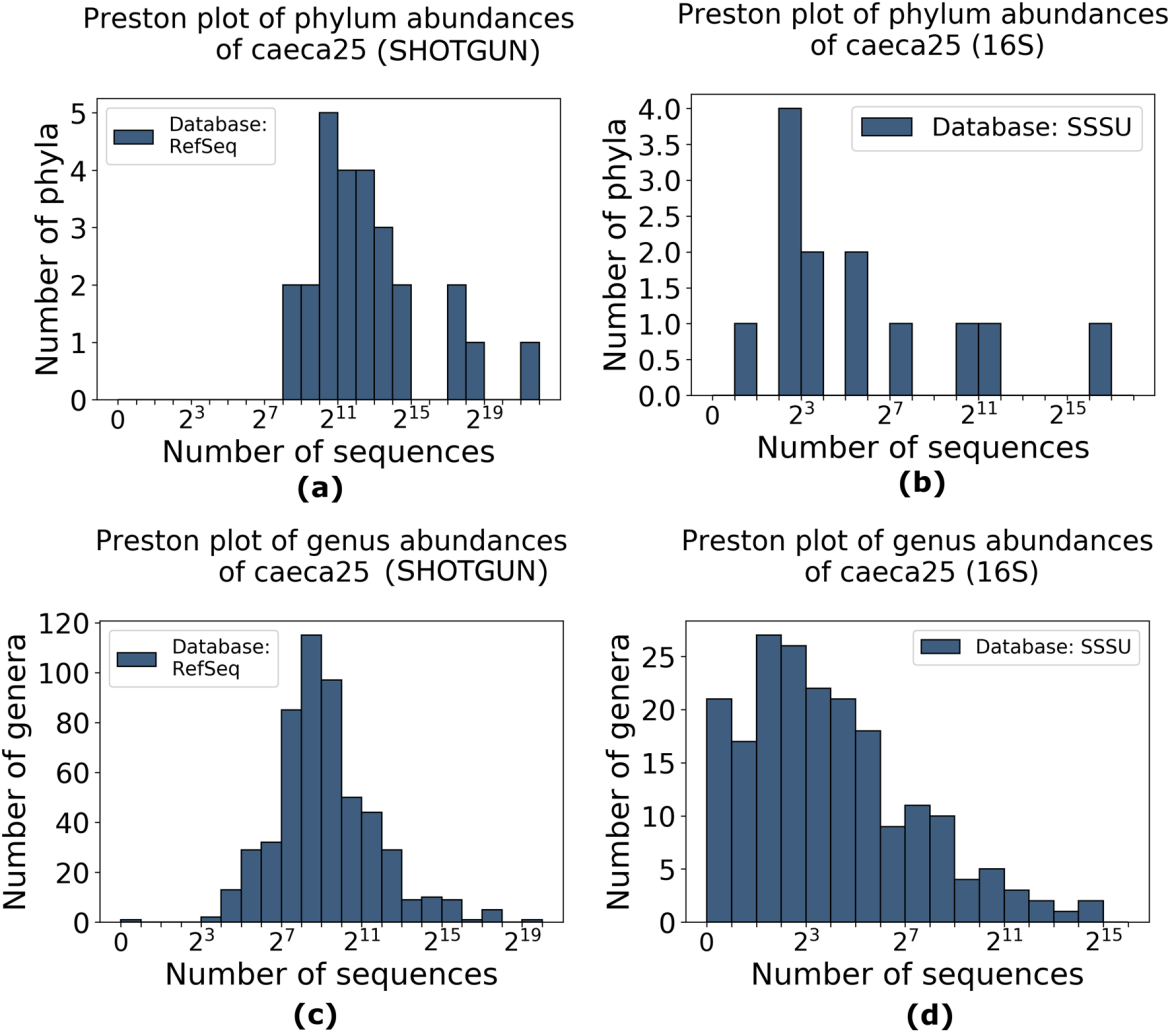
RSA histograms in logarithmic scale (Preston plots ^21^) of bacterial abundances in one sample selected as anexample (caeca25): (**a**) genera sampled by shotgun sequencing, (**b**) genera sampled by 16S rRNA sequencing, (**c**) phyla sampled by shotgun sequencing and (**d**) phyla sampled by 16S sequencing.

**Figure 2:**
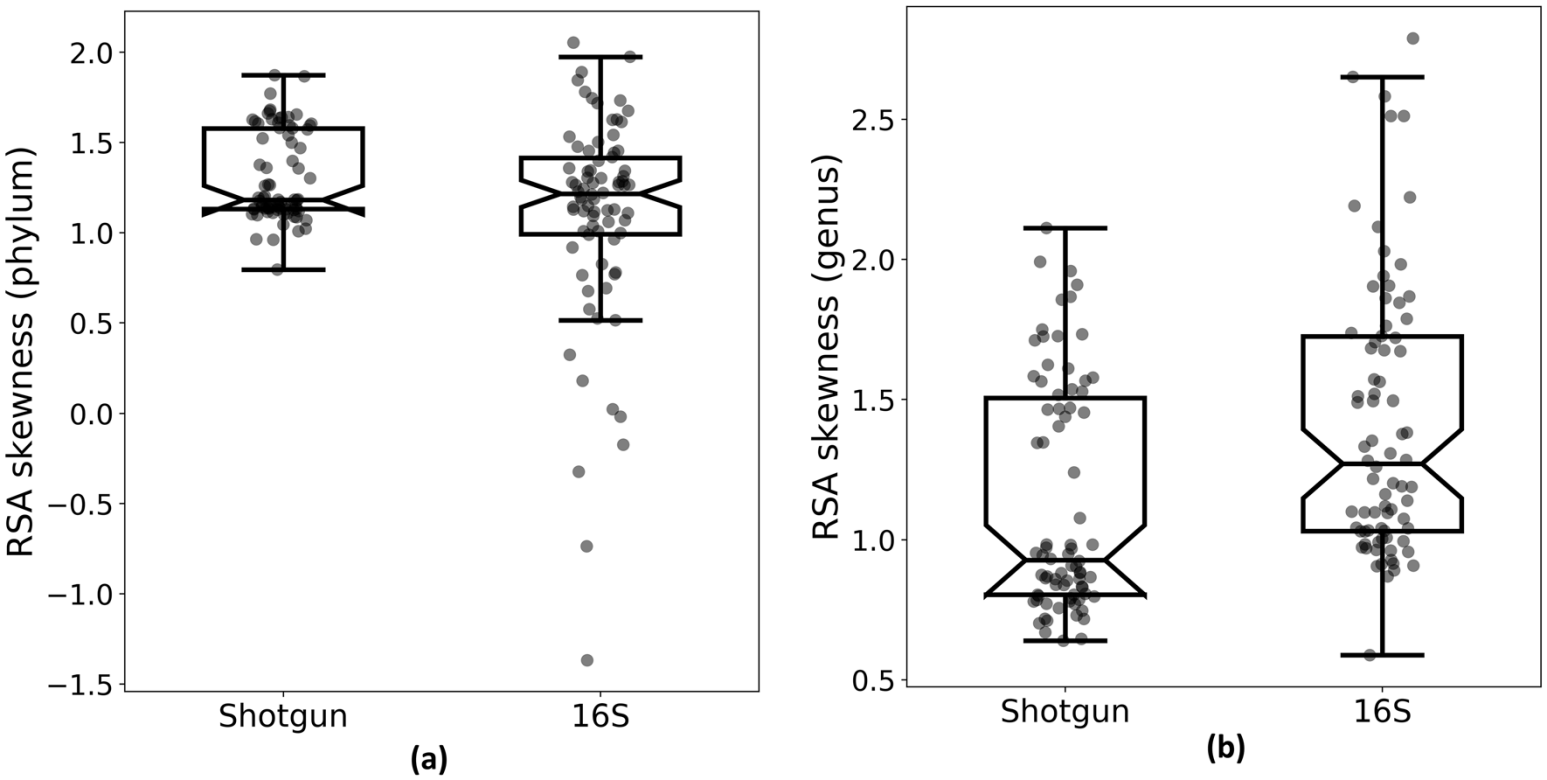
Box plot of the RSA skewness of bacterial communities at (**a**) phylum level and (**b**) genus level. Bacterial communities are sampled with (left) shotgun sequencing and (right) 16S sequencing.

In order to evaluate sample quality, we analysed both the Relative Species Abundance distribution (RSA) and the rarefaction curves. For each sample, we compared the RSA derived by shotgun and 16S sequencing. RSA histograms in logarithmic scale show that the distributions obtained by shotgun and 16S have similar shape at phylum level (Fig. 1a, b). In Fig. 1b, the 16S sample is characterized by a more patchy distribution, having identified less phyla. At phylum level, both strategies produce positively skewed samples in the log_2_-transformed distributions, except for 16S outliers, because none of the phyla is significantly rare (Fig. 2a).

On the other hand, at genus level, the two strategies display different shapes (Fig. 1c, d, Supplementary Fig. S1, S2, S3, S4). Indeed, the log_2_-transformed distributions derived by shotgun sequencing generally have a skewness closer to zero compared to those obtained by 16S, i.e. are more symmetrical (Figure 2b): a paired Student’s t-test on the skewness shows a significant difference between them (P = 8·10^–6^). This indicates that shotgun samples are characterized by a higher sampling size. According to Preston, left-skewed shapes of the RSA can be explained as artefacts of small sample size^21,22^, since insufficient sampling of the original space produces a truncation of the left tail of the RSA, increasing its skewness.

In shotgun samples, the RSA skewness at genus level is related to the total number of reads (Supplementary Fig. S5): the shotgun samples with the lowest total number of reads have the largest skewness. Specifically, Supplementary Figure S5 shows that shotgun samples cluster in two groups, one characterized by a low number of reads (# reads < 500,000, 28/78 samples) and a highly skewed RSA (greater than the 16S median), and one with a high total number of reads (# reads > 500,000, 50/78 samples) and a less skewed RSA.

Noticeably, the high-skewness group includes all 9 samples from 1st day, all 15 crop samples from 14th day and 4 out of 18 crop samples from 35th day. The samples collected at day 1 were very poor in terms of biomass and the crop samples contained more feed residues than caecal samples, making the DNA extraction less efficient both in terms of DNA quantity and quality. For the comparative analysis we removed samples with less than 500,000 reads being characterized by a low quality. This choice was corroborated by the analysis of the rarefaction curves, showing that shotgun samples with less than 500,000 reads do not reach a plateau in terms of identified genera (Supplementary Fig. S6). All the 50 samples included in the comparative analysis have a total number of reads > 500,000 and a skewness lower than the median of 16S samples, indicating a good sampling depth. Since included samples were characterized by a high microbial load, we are confident to extend the results of the following analyses only to samples with few contaminant DNA and low cross-contaminations. Nonetheless, we have shown that shotgun samples have a RSA similar to 16S samples when a low number of total reads is available, thus hypothesizing that in differential analyses carried on samples with a low microbial load regime, shotgun sequencing could perform similarly to 16S sequencing or even worse.

For a balanced comparison, also 16S samples corresponding to the discarded shotgun samples were removed.

### Differential analysis for the experimental conditions

**Figure 3:**
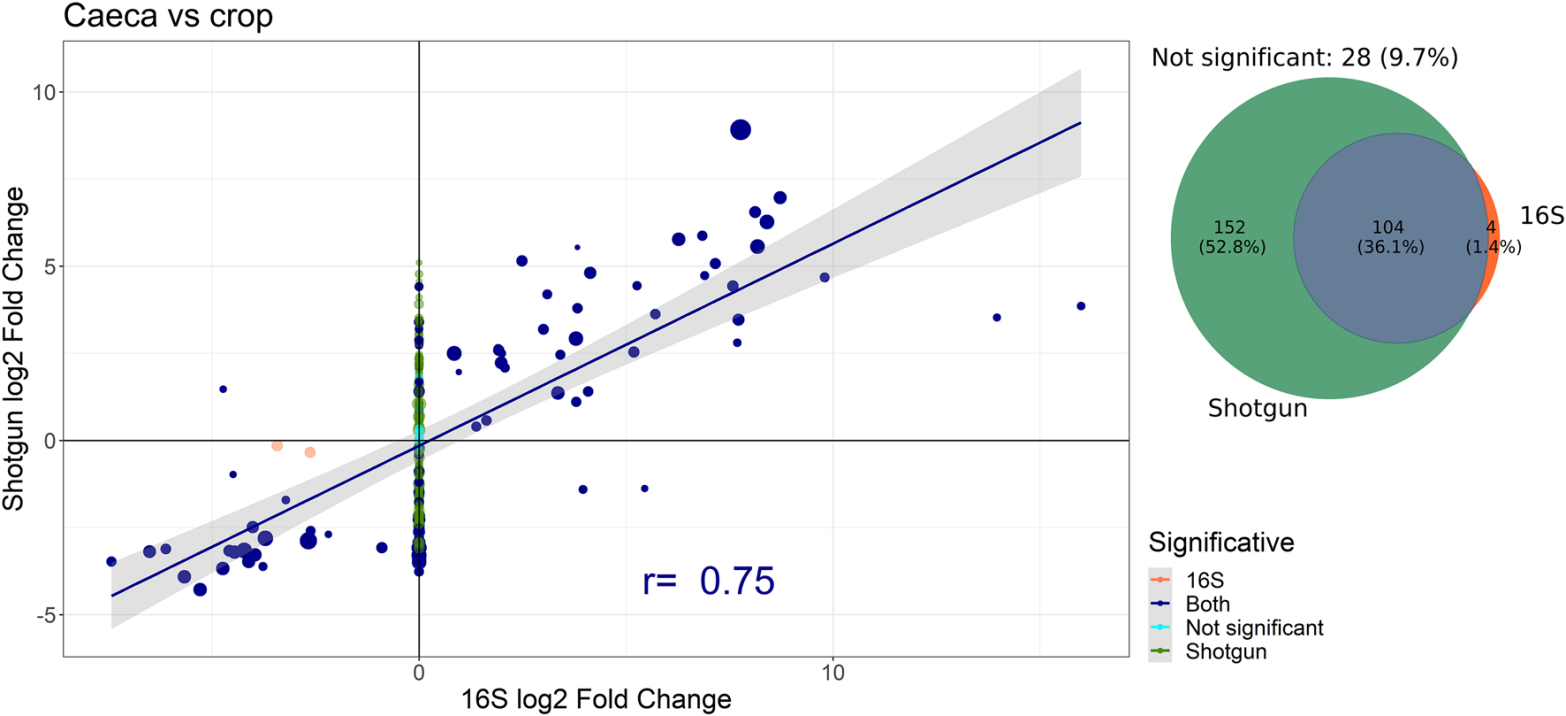
Fold changes between caeca and crop in genera identified by both strategies. Some fold changes are shrunk toward zero by the DESeq2 algorithm (see “Methods” section). Points with a statistically significant change for both strategies are represented in blue, for shotgun only in green, for 16S only in orange and without a significant change in cyan (adjusted P > 0.05 with DESeq2). Point size is the log_10_ of average number of reads from shotgun strategy mapping to each genus. Pearson’s correlation coefficient *r* and regression line are computed only on points with statistically significant fold changes according to both strategies (“Both” group in figure legend and in Table 1).

Since in many situations a metagenomic analysis is used to discriminate between different experimental conditions, we compared the results of differential analysis performed on reads obtained by the two strategies. To this aim, we analysed the fold changes of genera abundances between compartments of the GI tract and between sampling times (Fig. 3 for caeca vs crop, Supplementary Fig. S7 for 14th vs. 35th day) common to both sequencing strategies (288 genera for caeca vs crop, and 246 for 14th vs. 45th day). Comparing the genera abundances between caeca and crop, 16S identified 108 statistically significant differences (adjusted P < 0.05 with DESeq2), while shotgun identified 256; 28 genera, corresponding to 9.7% of total common genera, were not identified as significantly different by either strategy. Among the 104 genera identified to be different between caeca and crop by both sequencing strategies, 93.3% (97/104) showed a concordant fold change. Concerning the genera with different abundance between sampling times, 16S detected 58 statistically significant changes (adjusted P < 0.05 with DESeq2), while 75 were detected by shotgun. The 80% (16/20) of the 20 genera with different abundance between the 14th and 35th day for both sequencing strategies showed a concordant fold change (see Table). The discrepancies seem to be related to detection issues in 16S samples: indeed, all the seven discordant changes in caeca vs crop are caused by the partial or total absence of a genus in 16S samples (as shown in Supplementary Fig. S8). This is possibly due to the fact that these genera are close to the detection limit of 16S strategy, for which we have provided an estimate in the next section. On the other hand, three of the four discordant genera in the 14th vs 35th day showed actual discrepancies between shotgun and 16S changes, not necessarily caused by detection issues (see Supplementary Fig. S9).

**Table 1:** Percentage of genera having a concordant change in abundance in shotgun and 16S samples with a statistically significant change for: both sequencing strategies (1st column), 16S only (2nd column), Shotgun only (3rd column), neither (4th column).

	16S & shotgun	16S only	Shotgun only	Not significant
Caeca versus crop	93.3 (97/104)	75.0 (3/4)	72.4 (110/152)	64.3 (18/28)
14th versus 35th day	80.0 (16/20)	55.3 (21/38)	58.2 (32/55)	57.1 (76/133)

Noticeably, shotgun sequencing found 152 statistically significant changes in genera abundance between caeca and crop of chickens that 16S sequencing failed to detect, while 16S found only 4 changes that shotgun sequencing did not identify (Fig. 3).

### Genera detection and abundance quantification

**Figure 4:**
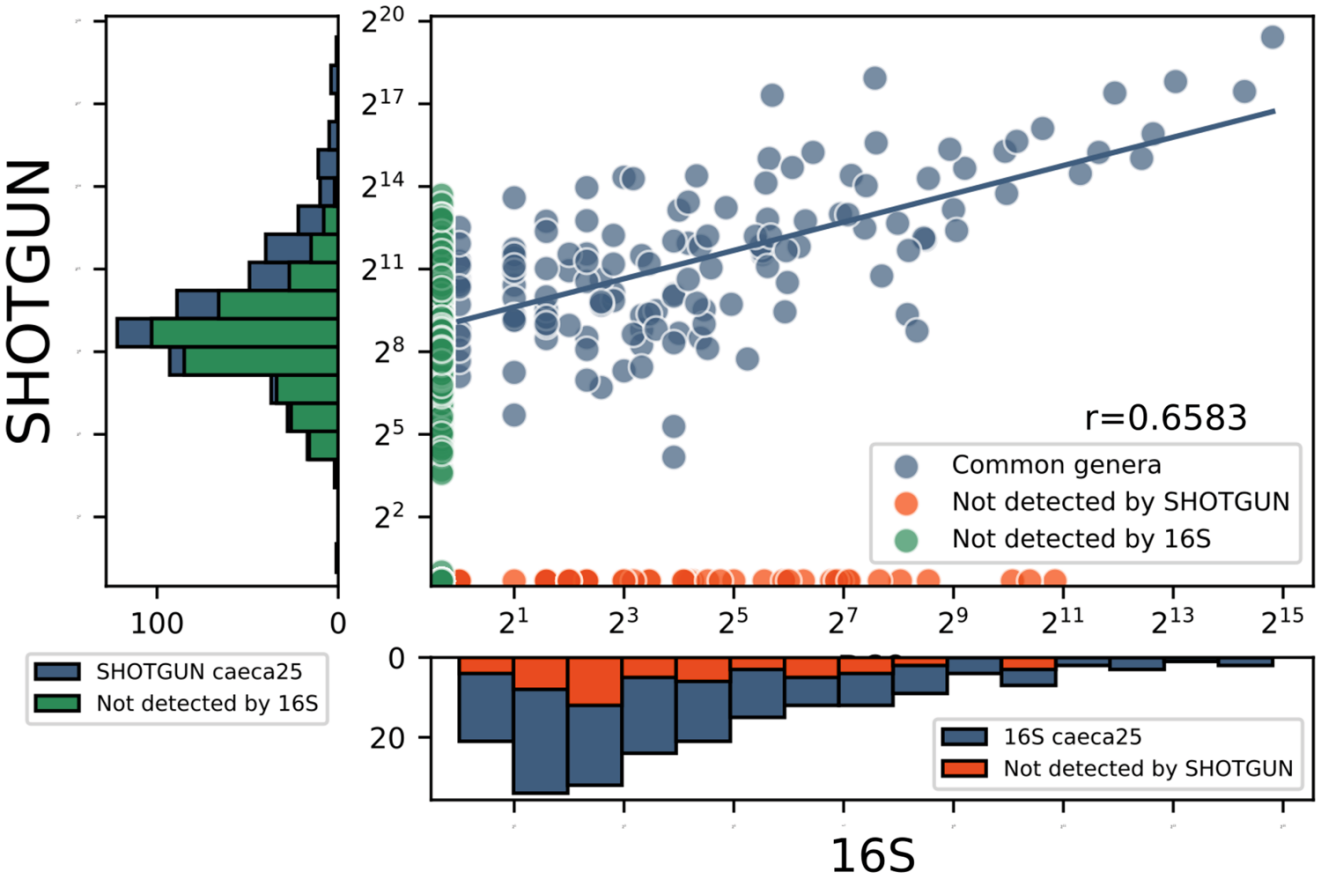
Scatter plot of 16S and shotgun genera abundances of one sample selected as example (caeca25). Histograms display stacked bars, where every column is divided in a part corresponding to the abundance of genera detected by both sequencing strategies (blue) and the other part is relative to genera detected exclusively by only one strategy (red for 16S and green for shotgun). Pearson's correlation coefficient is computed only for the common genera. Logarithmic (log_2_) scale helps to recognize that less abundant genera identified by shotgun sequencing are almost not detected by 16S sequencing.

The agreement between the taxonomic profiles estimated with the two strategies was further evaluated computing for each sample the Pearson’s correlation coefficient (r) between the taxonomic abundances of genera common to 16S and shotgun sequencing. Overall, we observed a good agreement between the taxonomic abundances found by the two strategies (Fig. 4, Supplementary Fig. S10), with an average correlation of 0.69 ± 0.03 in caeca (all p-values < 10^–11^ for correlation significance between 16S-shotgun sample pairs) and 0.75 ± 0.05 in the crop (all p-values < 5·10^–5^).

**Figure 5:**
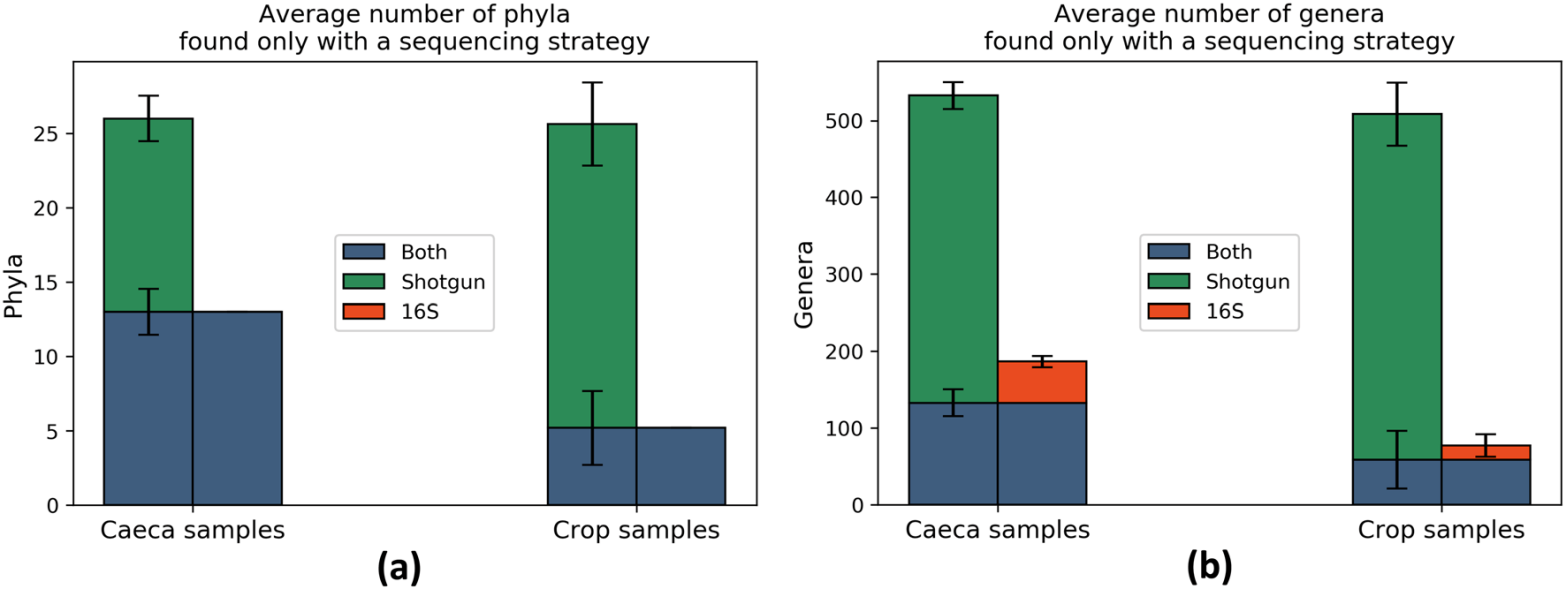
Average number of (**a**) phyla and (**b**) genera found within caeca and crop samples. The length of the error bars is equal to the standard deviation computed on all the samples.

A larger difference is observed between the number of identified taxa by the two strategies. The histogram on the left of Fig. 4 shows that, in the selected sample, a great majority of genera detected by shotgun sequencing are not found by 16S sequencing (green portion of the bars). This phenomenon is mostly observed on the leftmost bins, that represent low abundance genera (Supplementary Fig. S11, S12, S13, S14 for single samples). These results are confirmed in Fig. 5 and Supplementary Table S1, showing that (a) all the phyla detected in 16S samples were identified in shotgun samples and (b) only a small number of genera (about 23% for caeca samples and 11% for crop) is recovered by both strategies. The percentage of reads mapping to the genera identified by both sequencing strategies is large (on average 89% for caeca and 99% for crop). This means that genera identified by both strategies are the most abundant ones, e.g. those mapped by most of the reads. In summary, shotgun sequencing always identifies more taxa than 16S sequencing, as also reflected on the rarefaction curves (Supplementary Fig. S6). Moreover, 16S sequencing predominantly detects taxa that are also identified by shotgun sequencing (133 of 187 genera and 14 of 14 phyla on average) (Fig. 5).

**Figure 6:**
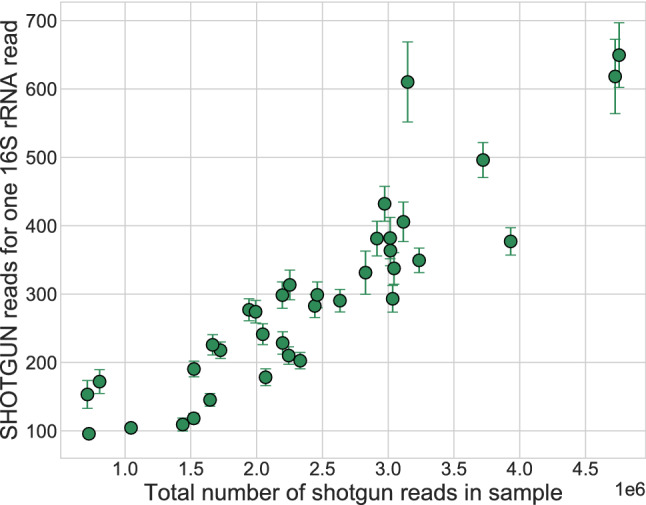
Intercepts of shotgun vs 16S abundance linear regressions of caeca samples against the total number of reads in each set, representing the number of shotgun sequences corresponding to one 16S sequence. Error bars correspond to the confidence interval for the parameter provided by the fit.

The relationship between abundances detected by 16S and shotgun metagenomics was further investigated fitting a linear regression model on the abundance of genera common to both strategies in each sample. We considered, for each shotgun-16S pair of samples, the logarithmic abundances obtained with 16S as independent variable and those obtained with shotgun as dependent variable, so that the intercept in this model represents the number of shotgun reads corresponding to genera that are mapped to one single read by 16S sequencing, that we consider as a detection limit. Here, samples with low number of reads (< 500,000) were included for completeness. Results show that the model intercept increases as a function of the total number of reads available in shotgun samples (Fig. 6). The regression intercept and the total number of shotgun reads are positively correlated (Pearson’s coefficient = 0.93, P < 10^–16^).

Hence, given the total number of reads of a shotgun sequencing sample, the genera mapped by a number of reads lower than the model intercept are the most likely to be undetected by 16S strategy. For example, considering the sample depicted in Fig. 4 with a total number of reads equal to 2,972,671 with shotgun strategy, genera with a number of reads approximately < 350 would probably not be detected by 16S sequencing, corresponding to 88% of total detected genera in that shotgun sample.

### Sample stratification by experimental condition

Since in many studies an unsupervised analysis (i.e. clustering) based on taxonomic profiling is performed, in order to identify possible sample stratification due to experimental conditions or to other unknown factors, we calculated the Bray–Curtis beta diversity and performed a Principal Coordinate Analysis (PCoA). We considered for every sample a n-dimensional (n = 678) vector of abundances, considering the genera that are common to all samples (a graphical representation is showed in Supplementary Figure S15). A quantitative evaluation of the separation in the PCoA space of samples labelled by experimental conditions was obtained through the mean Silhouette Score (SS) of the samples at genus level. We compared the silhouette scores either on the totality of genera identified by shotgun (“SHOTGUN”) or 16S (“16S”) strategy, or on the subset of genera detected exclusively by shotgun (“SHOTGUNex”) or by 16S (“16Sex”), to evaluate their ability to discriminate between known experimental conditions (compartment of the GI tract and sampling time). Results show that when samples are labelled according to the compartment of the GI tract, the Silhouette Score is high (i.e., close to 1) for both strategies (see Supplementary Tab. S2 and Supplementary Fig. S16). However, the separation between groups is stronger in shotgun samples than in 16S samples. This result remains true even when the PCoA of shotgun samples is computed considering only shotgun-specific genera.

**Figure 7:**
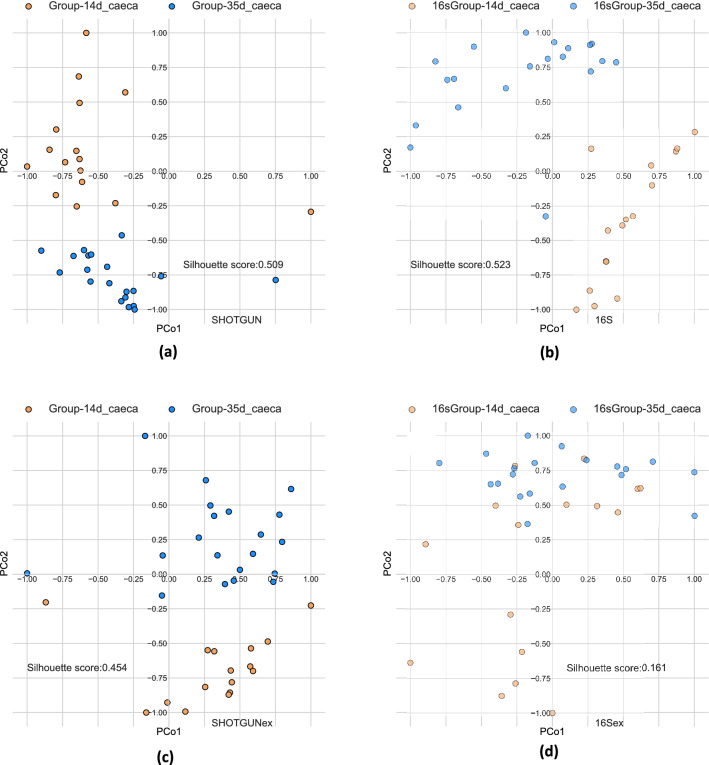
PCoA based on the beta-diversities between samples (Bray–Curtis metric), computed on genera abundances of caeca samples normalized by DESeq2, labelled by sampling time: 14th day (gold), 35th day (cyan). (**a**, **b**) with all genera detected respectively by shotgun (**a**) and 16S (**b**); with genera found exclusively by shotgun (**c**) or 16S (**d**).

On the other hand, when grouping samples according to sampling time within the same compartment of the GI tract, Silhouette Scores are generally lower for both strategies, while still achieving a good separation (see Fig. 7). Figure 7 plots refer to the Silhouette Scores of Supplementary Table S3, in which full samples (Fig. 7a,b) reach an almost perfect separation, though the low compactness leads to worse scores with respect to GI tract labelling. An interesting result is that abundance profiles of genera found only in shotgun sequencing (SHOTGUNex) have a positive silhouette score, while 16Sex samples have a smaller silhouette score, close to 0 (a value representing low-quality clustering, very close to random). This result highlights that shotgun sequencing detects low-abundance genera that carry significant information about the experimental biological factors, while 16S-specific genera fail to correctly cluster samples based on one of the experimental factors considered (namely sampling time), and a good separation is obtained only when the most abundant genera common to both strategies are considered.

## Conclusion

This comparative analysis, based on 78 chicken gut metagenomes, showed that shotgun sequencing recovered more information about low-abundance genera than 16S sequencing, when a sufficient number of reads were available for taxonomic profiling (> 500,000 reads). Most of the bacterial genera were identified by shotgun sequencing, while, on average, 16S recovered less than 31% of the genera and less than 50% of the phyla.

In agreement with our study, Campanaro et al. (2018)^17^ showed that several phyla were strongly under-represented in the 16S amplicon analysis in comparison to random shotgun DNA sequencing. Moreover, Laudadio et al. (2018)^16^ highlighted the higher resolution of taxonomic analyses performed by shotgun metagenomics as compared to 16S sequencing at different taxonomic levels, using the number of taxa identified in each sample as a metric to evaluate performance. Tessler et al. (2017)^23^ and Shah et al. (2011)^24^ showed instead that 16S rRNA sequencing identified more diverse bacterial phyla and families than shotgun sequencing, but we remark that in both studies the number of reads per sample used for taxonomic profiling was about 100 time smaller than in our study and in the two previously mentioned papers, thus this undersampling might be the reason of this apparent inconsistency (see Supplementary Table S4). In our work, we demonstrated that the total number of sequenced reads is a critical factor to perform a robust analysis with shotgun sequencing at low taxonomic (genus) level. Furthermore, analysing the changes in genera abundance due to different experimental conditions (i.e., different compartments of the GI tract and sampling time), we showed that shotgun sequencing is more sensitive than 16S, being able to identify a larger number of genera that are significantly affected by these factors (Fig. 3).

The RSA distributions obtained by the two sequencing strategies showed quantitative and qualitative differences at genus level, in particular for the left tail. When the total number of reads was as low as ~ 200,000 in shotgun samples, the RSA shapes became strongly positively skewed, similarly to those obtained with 16S. On the other hand, increasing the sampling intensity enabled to detect less abundant genera^21^. The genera detected only by shotgun sequencing are not very abundant, because even if they constitute about 75% of the identified genera, the associated read count is less than 9% of the total reads. Nonetheless, these less abundant genera provided reliable information and showed significant correlation with the different experimental conditions. The same conclusion could not be drawn for low-abundance genera exclusively identified by 16S sequencing. The latter were able to discriminate different compartments of the GI tract but failed to stratify samples according to the sampling time. Finally, our results allowed to provide an estimation of the number of genera that would likely be detected by shotgun sequencing and not by 16S sequencing.

## Methods

### Animals and treatments

The experimental trial from which the samples were collected has been previously described in De Cesare et al. (2020)^20^. In our study, we compared bacterial abundance profiles in 78 samples undergoing both 16S and shotgun metagenomic sequencing. Overall, 40 samples were from caeca (4 from 1st day, 16 from 14th day and 20 from 35th day) and 38 from crop (5 from 1st day, 15 from 14th day and 18 from 35th day) were collected.

Shotgun sequencing was performed as previously described^20^, whereas for amplicon sequencing, the libraries were prepared following the Illumina 16S Library preparation protocol^10^, amplifying the variable V3 and V4 regions of the 16S rRNA. Sequencing was performed in paired-end at 150 bp in the Illumina MiSeq^25,26^. The maximum output of the v2 kit is 15 million reads per run, meaning approximately 187,500 reads per sample.

Since biomasses extracted from GI tracts (caecum in particular) have typically a high microbial load^27^ and host depletion methods can be not effective for DNA libraries^28^, the samples were not depleted from host DNA. The reads not assigned to Bacteria domain were hence removed after taxonomic profiling.

Overall, 78 metagenomes were analysed: 40 metagenomes from caeca (i.e., 4 from 1st day, 16 from 14th day and 20 from 35th day) and 38 from crop (i.e., 5 from 1st day, 15 from 14th day and 18 from 35th day). The 16S and shotgun metagenomes analysed as well as their number of sequences mapping to Bacteria are detailed in Supplementary Table S5, with genera rarefaction curves in Supplementary Figure S6.

### Processing and taxonomic profiling

Both 16S and shotgun raw reads were pre-processed and assigned to a genus using MG-RAST with default parameters^29^. Specifically, 16S reads were taxonomically classified using the Silva SSU reference database^30^, while the RefSeq database^31^ was used for shotgun reads. Singleton reads were discarded.

MG-RAST advices against the reliability of taxonomic profiling at species level^29^, in particular for samples obtained by shotgun sequencing. For this reason, we choose to perform the analysis of the bacterial communities up to genus level.

### Statistical analysis methods

Data were processed and visualized in Python 3.6 and R 3.6.0 using custom scripts.

For each sample, the Relative Species Abundance distribution (RSA) was computed counting the number of genera that have a certain abundance. RSAs were visualized as Preston plots^21,32^. Rarefaction curves were computed with an extension^33^ of the R package phyloseq ^34^ implementing the package vegan^35^.

Counts normalization and differential genera abundance analysis were performed by DESeq2 package^36^, considering as significant those changes with an adjusted p-value lower than 0.05. High variability in the dispersion of the counts was adjusted with a shrinkage procedure by DESeq2, that led to smaller estimates of the fold changes in samples with a low number of total reads. For each sample, we calculated and visualized the Pearson’s correlation coefficient between the taxonomic abundances obtained using 16S and shotgun, along with a p-value for correlation significance. For this analysis, we considered only genera that were present in both profiles.

Beta diversity was computed using the Bray–Curtis distance^37,38^ and considering normalized counts retrieved by DESeq2, that keeps into account the differences in sample size. Principal Coordinate Analysis (PCoA)^39^ was performed to visualize the samples based on the beta diversity. Silhouette Scores were calculated to assess the correspondence between sample displacement in the PCoA space and experimental factors (i.e. compartments of gastrointestinal tract and sampling time). Sampling time corresponds to the days of rearing of the chickens (1st, 14th and 35th).

### Ethical approval and informed consent

The experiments were conducted after obtaining the approval of Ethical Committee of the University of Bologna on 17/3/2014 (ID: 10/79/2014). All experiments were performed in accordance with relevant guidelines and regulations.

## Supplementary Information

Supplementary Information.

## Data Availability

All 16S and shotgun metagenomic sequences tested as part of this comparative study were deposited in MG-RAST (http://metagenomics.anl.gov/) and are public available under the projects named newlacto16S (http://www.mg-rast.org/mgmain.html?mgpage=project&project=mgp91466) and newlacto (http://www.mg-rast.org/mgmain.html?mgpage=project&project=mgp13081), respectively.
