# Supplementary material for: Comparison between 16S rRNA and shotgun sequencing data for the taxonomic characterization of the gut microbiota
Source: Sci Rep. 2021 Feb 4;11:3030. doi: 10.1038/s41598-021-82726-y (PMC7862389; doi:10.1038/s41598-021-82726-y)
Supplement: Supplementary file 1 — Supplementary Information. [file 41598_2021_82726_MOESM1_ESM.pdf]

Supplementary information

Comparison between 16S rRNA and shotgun sequencing data for the taxonomic characterization of the gut microbiota

Francesco Durazzi[1], Claudia Sala[1], Gastone Castellani[2], Gerardo Manfreda[3], Daniel Remondini[1], Alessandra De Cesare[4]

[1]Department of Physics and Astronomy, University of Bologna, Bologna, 40127, Italy
[2]Department of Experimental, Diagnostic and Specialty Medicine – DIMES, University of Bologna, Bologna, 40127, Italy
[3]Department of Agricultural and Food Sciences, University of Bologna, Ozzano dell'Emilia, 40064, Italy
[4]Department of Veterinary Medical Sciences, University of Bologna, Ozzano dell'Emilia, 40064, Italy

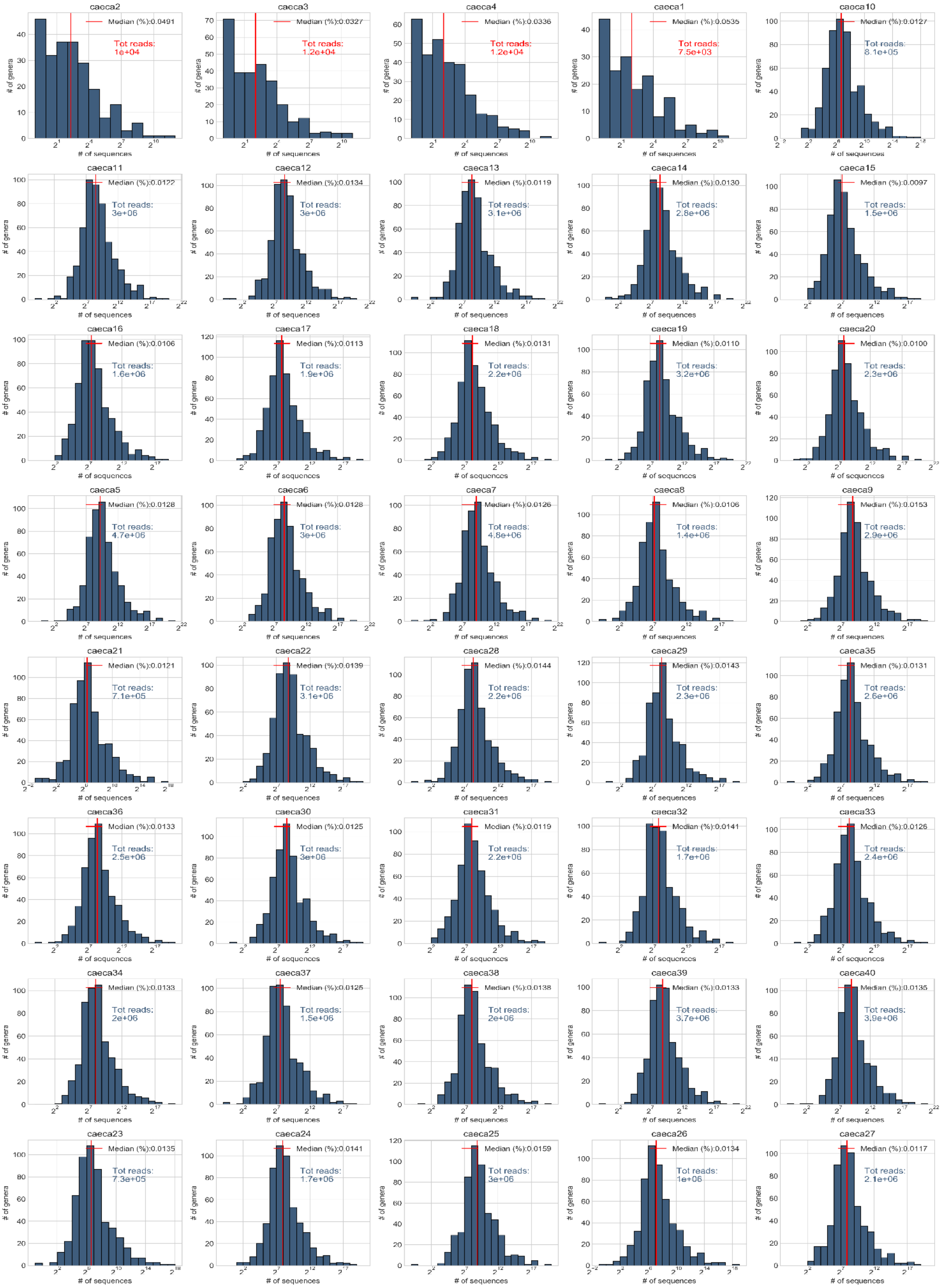

Figure S1. Preston plot of genera abundances in all shotgun samples from caeca. Median is shown as percent genus abundance. Number of reads written in blue are those above the threshold that developed a left tail (500000), while those written in red are below it (low coverage).

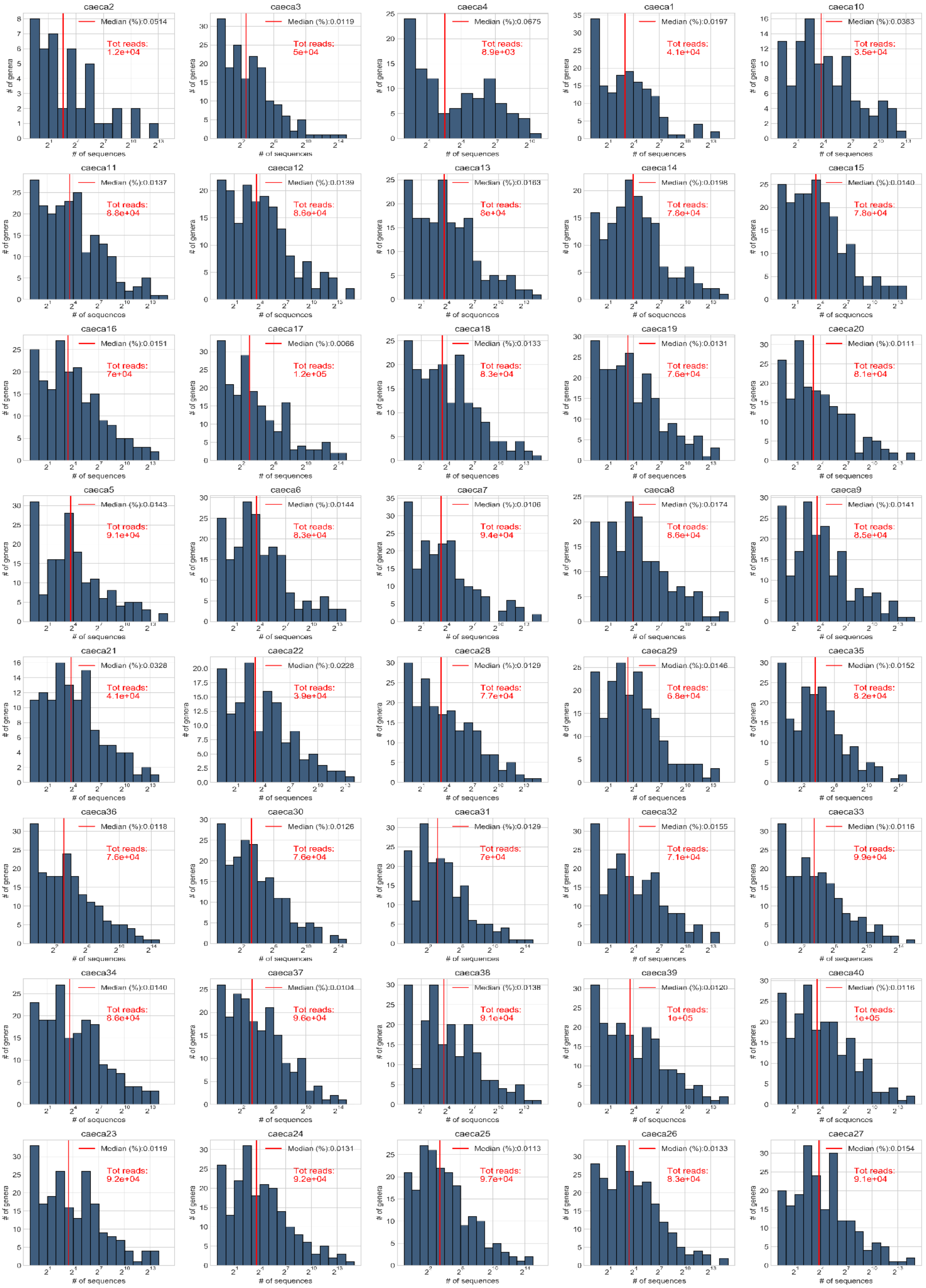

Figure S2. Preston plot of genera abundances in all amplicon samples from caeca. Median is shown as percent genus abundance.

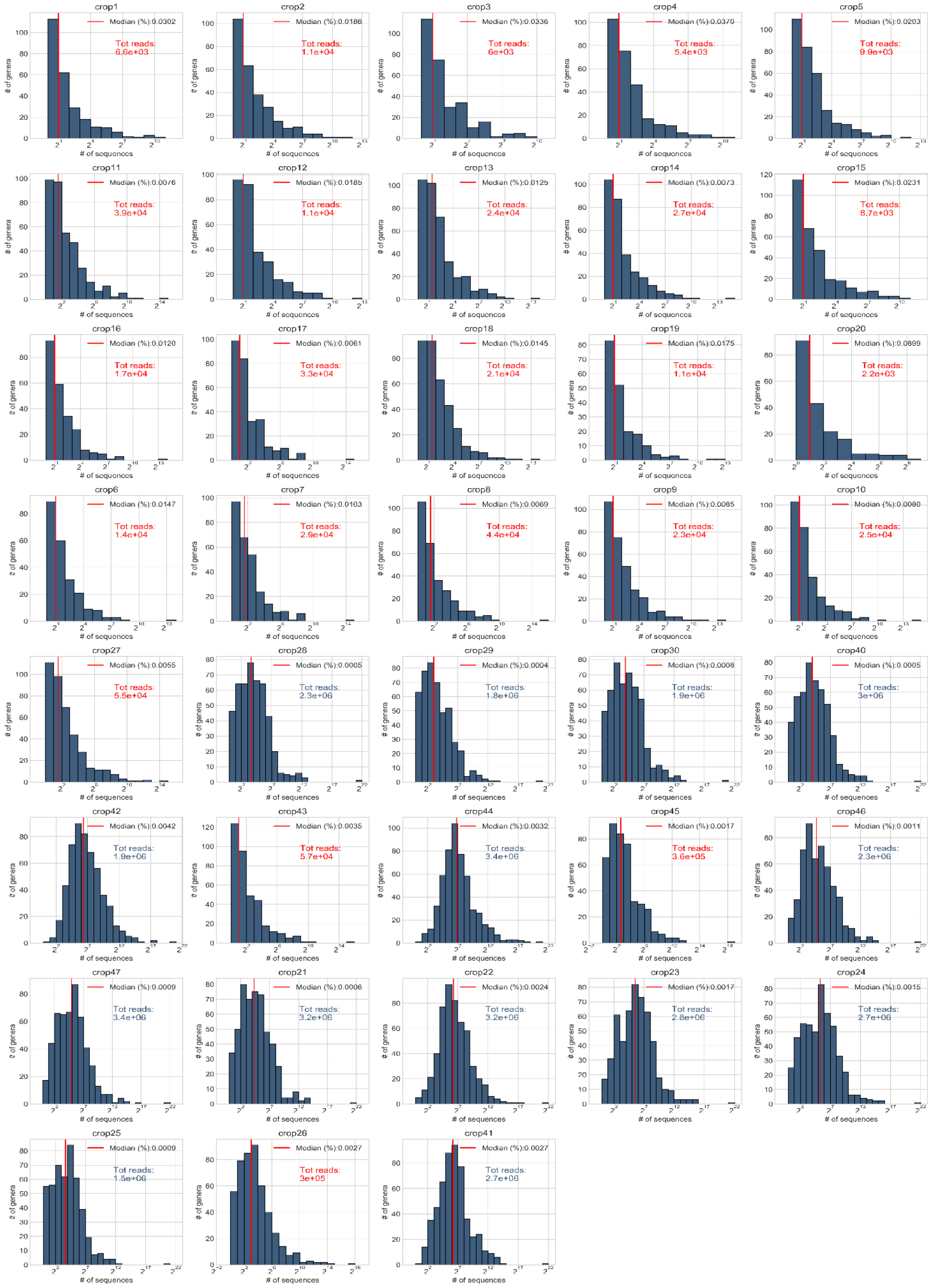

Figure S3. Preston plot of genera abundances in all shotgun samples from crop. Median is shown as percent genus abundance. Number of reads written in blue are those above the threshold that developed a left tail (500000), while those written in red are below it (low coverage).

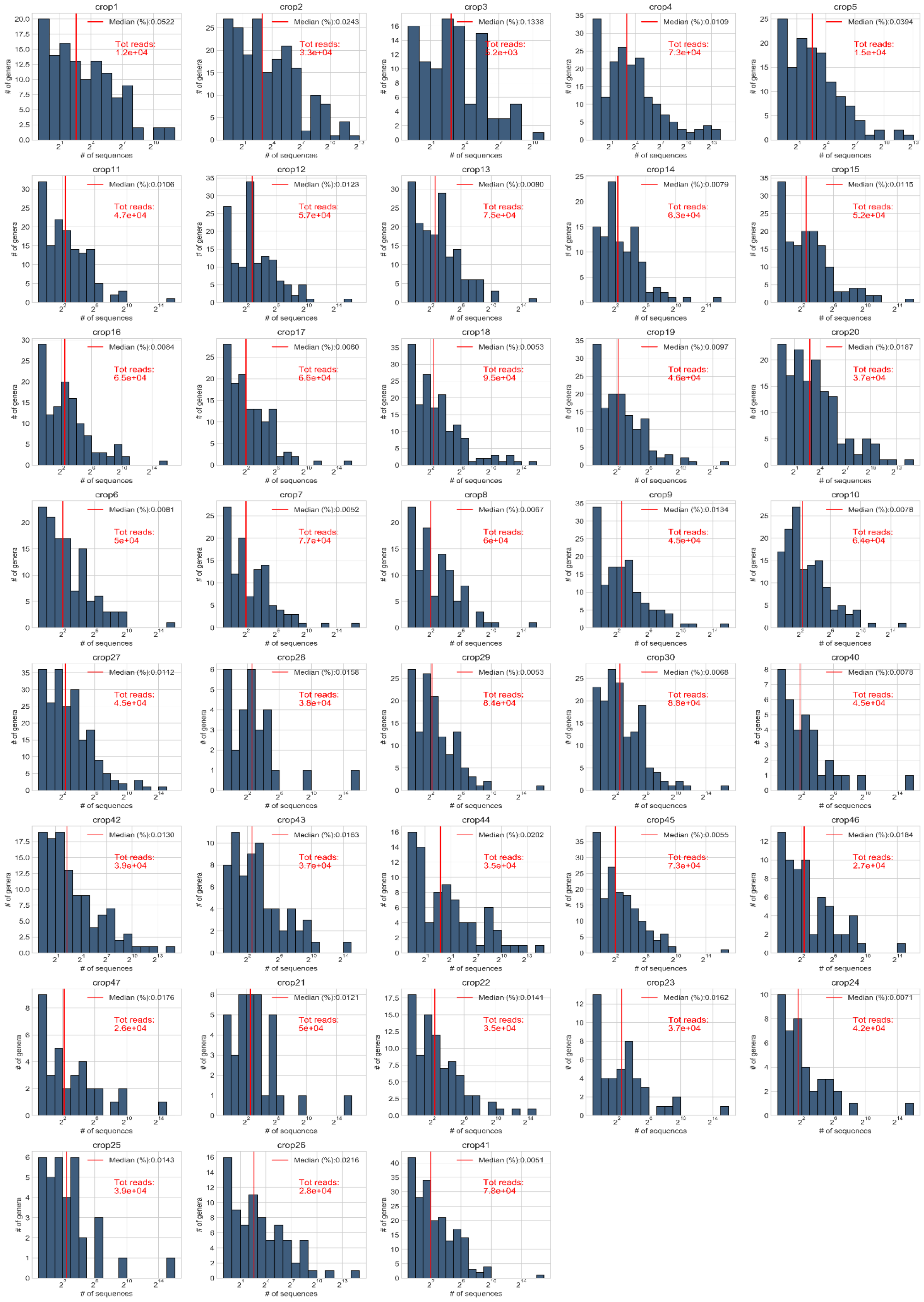

Figure S4. Preston plot of genera abundances in all amplicon samples from crop. Median is shown as percent genus abundance.

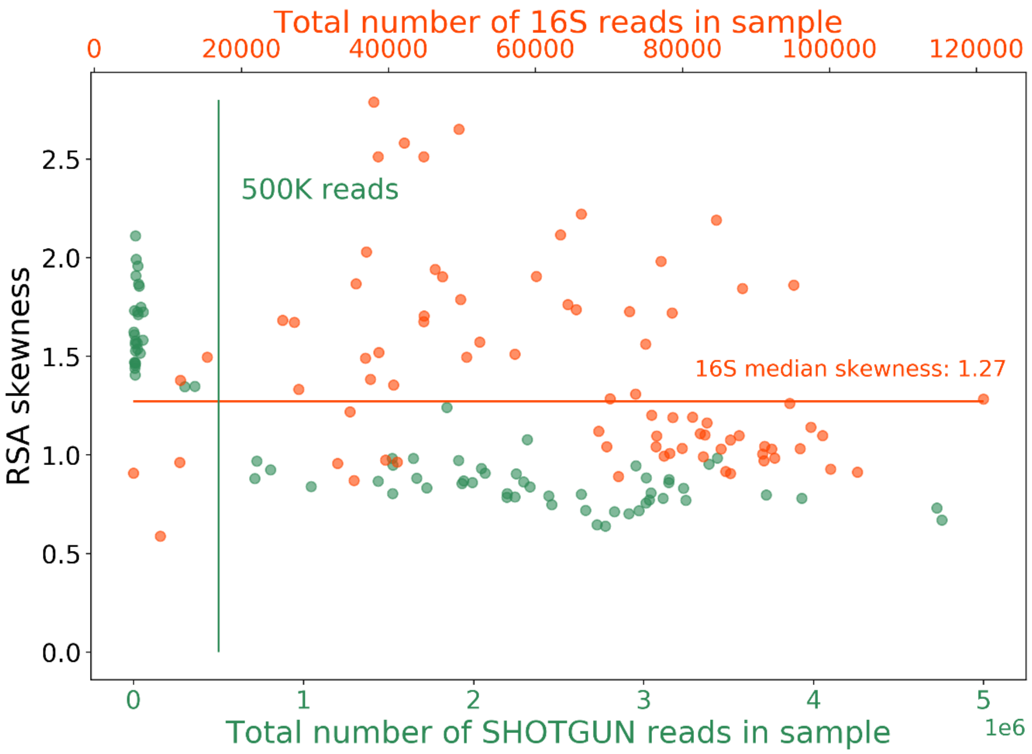

Figure S5. Skewness of abundance profiles. Skewness of abundance profiles versus the total number of reads in shotgun samples (GREEN) and in 16S samples (RED). On average, 16S samples have bigger skewness, except for low total read number (<500000) in shotgun samples, i.d. on the left of the green vertical line. All the 50 shotgun samples having more than 500000 reads, have lower skewness than 16S median value (orange horizontal line), i.e. have a more symmetric RSA.

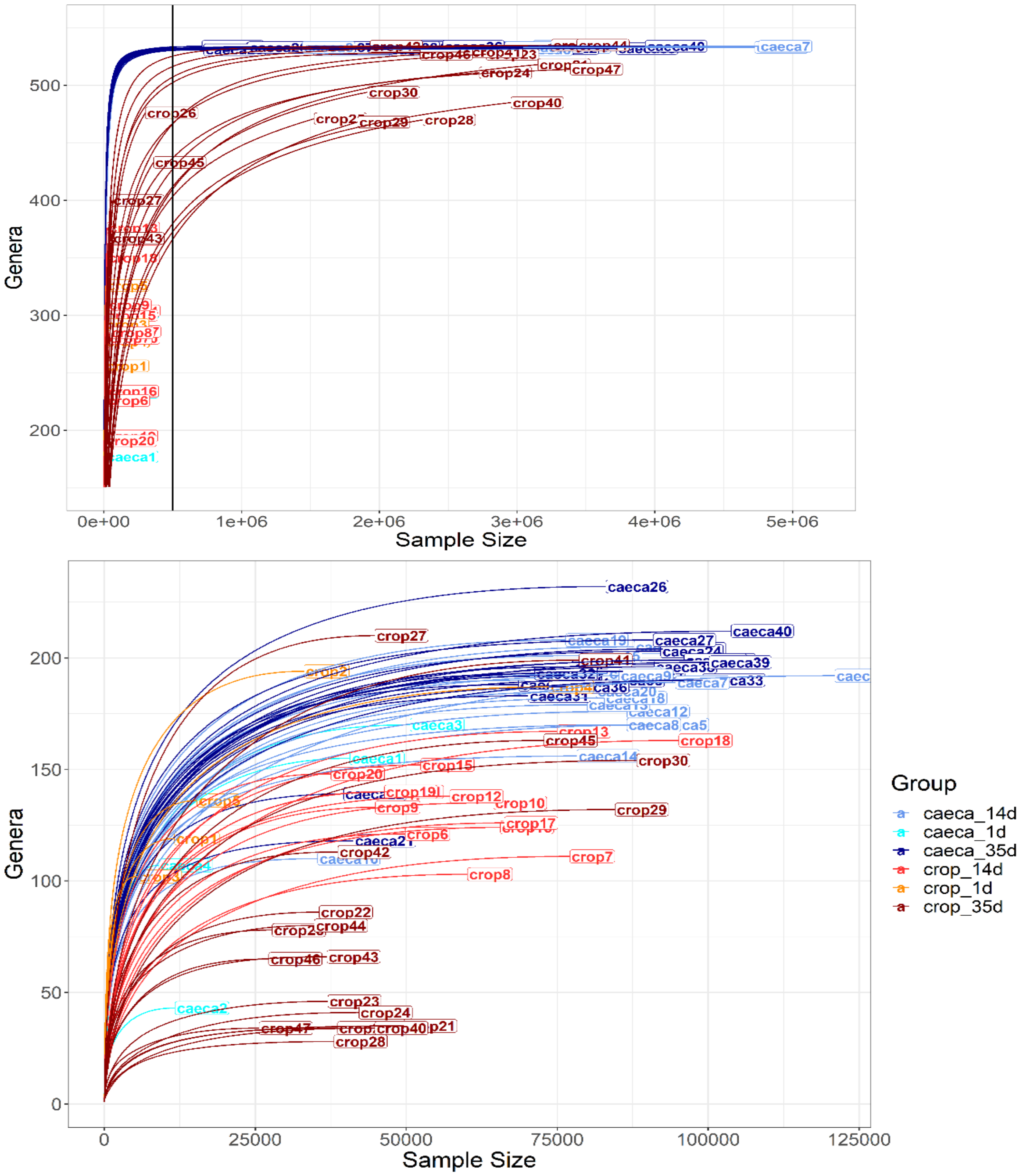

Figure S6. Genera rarefaction curves. (Top) shotgun samples and (Bottom) 16S samples. On the x-axis: sub-sampling of the number of reads. On the y-axis: number of bacterial genera identified in the sub-samples of reads.

Top: shotgun sequencing detects more taxonomical richness (177-535 genera) than 16S samples (28 – 232 genera). Samples not reaching the plateau in terms of identified genera, have less than 500000 mapped reads, so rarefaction curves end on the left of the black vertical line for these samples.

Bottom: the rarefaction curves of all the 16S samples reach the plateau but the total number of genera identified by 16S sequencing results lower than the one identified by shotgun sequencing.

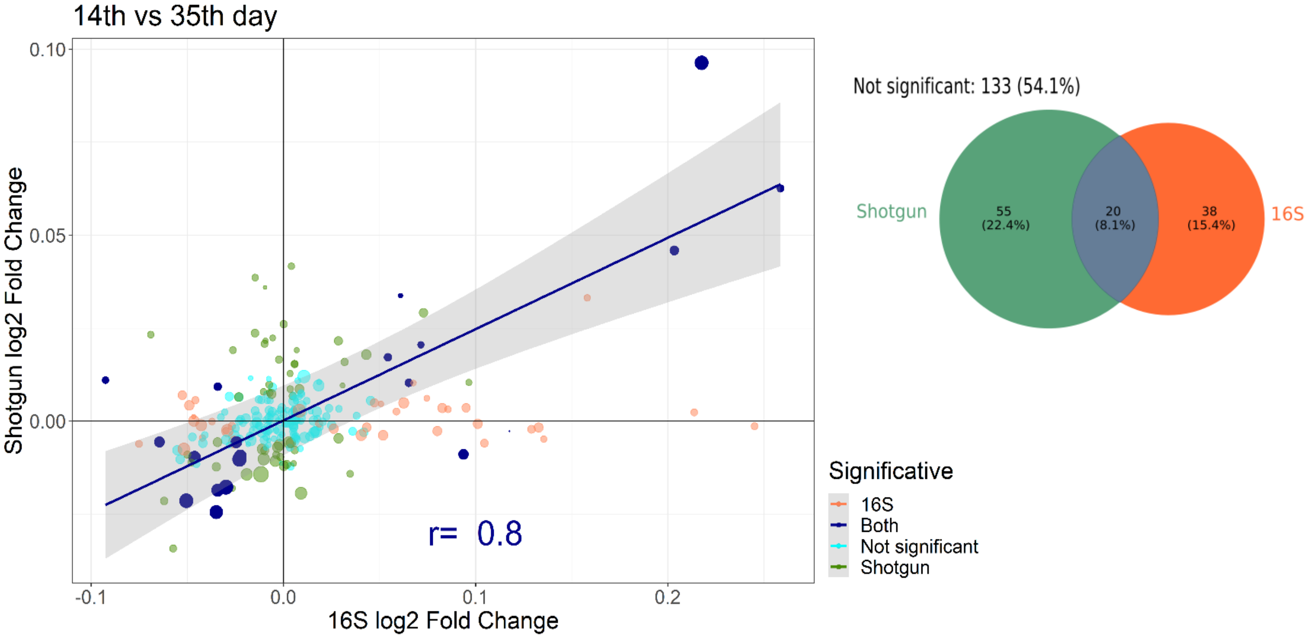

Figure S7. Fold changes between 14th and 35th day in genera identified by both strategies. Some fold changes are shrunk toward zero by the DESeq2 algorithm (see Material and Methods). Points with a statistically significant change for both strategies are represented in blue, only for shotgun in green, only for 16S in orange and without a significant change (adjusted p-value>0.05) in cyan. Point size is the log_{10} of average number of reads from shotgun strategy mapping to each genus. Pearson's correlation coefficient (r) and regression line are computed only on points with statistically significant fold changes according to both strategies ("Both" group in figure legend and in Table 1).Point size is the log_{10} of the base mean of number of reads mapping to each genus-point in shotgun samples. Pearson's correlation coefficient (r) is computed only for "Both" group.

Genus	Shotgun genera abundances	16S genera abundances
**Desulfomicrobium**	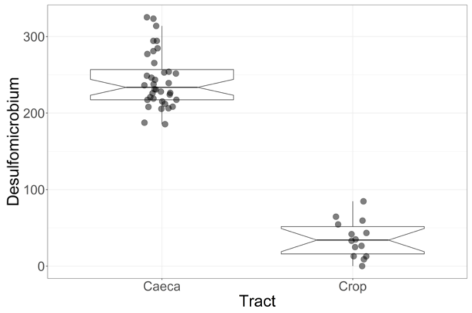	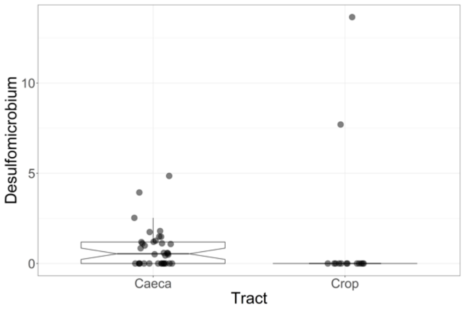
**Halanaerobium**	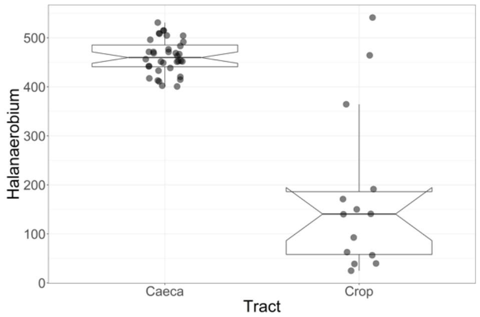	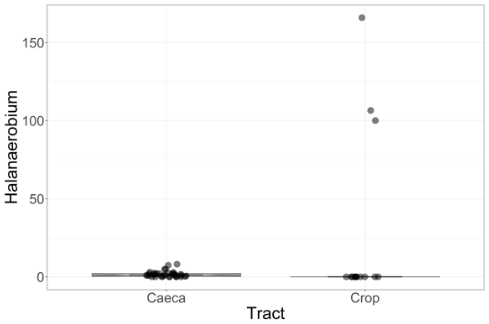
**Lyngbya**	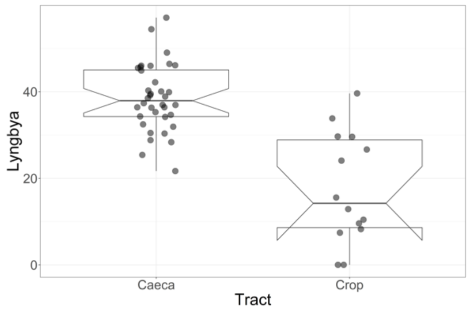	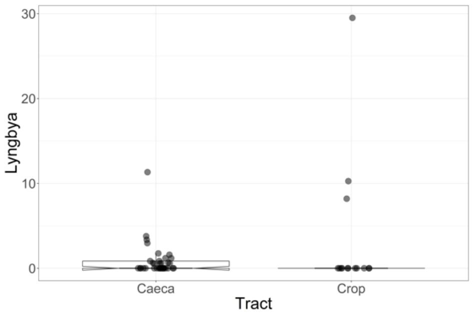
**Microcystis**	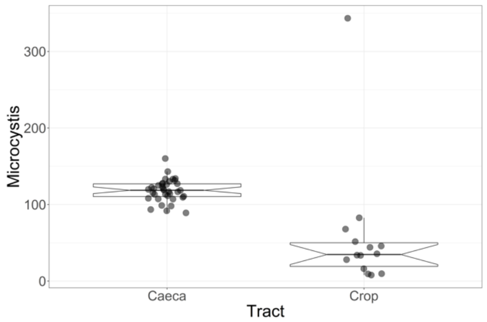	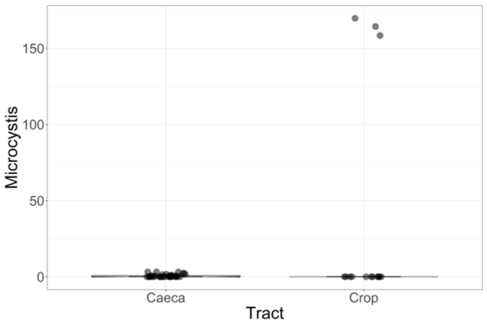
**Oscillatoria**	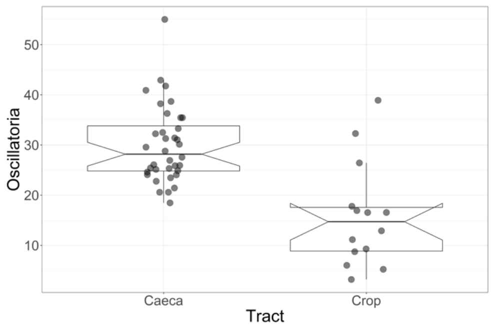	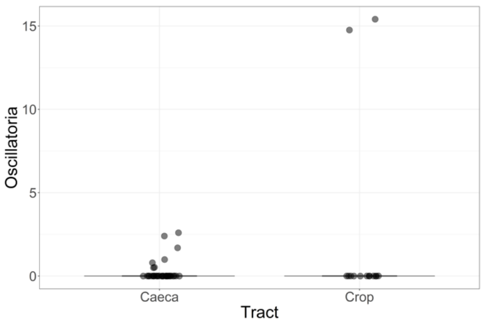

**Synechoccus**	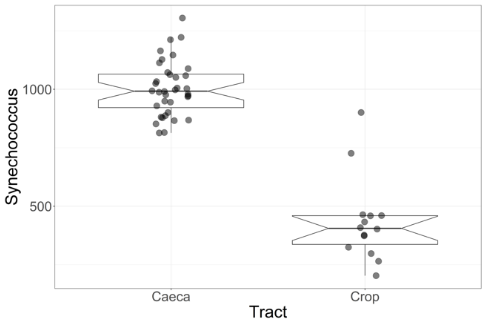	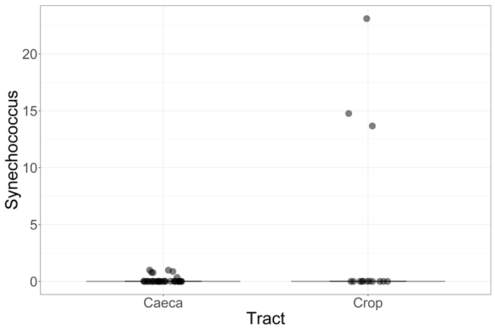
**Stackebrandtia**	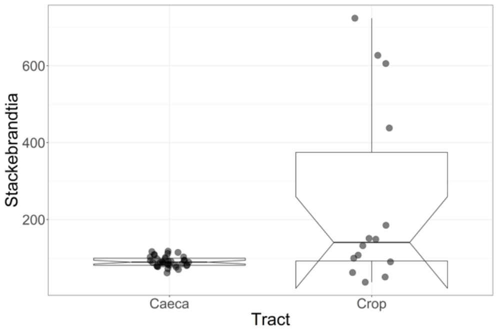	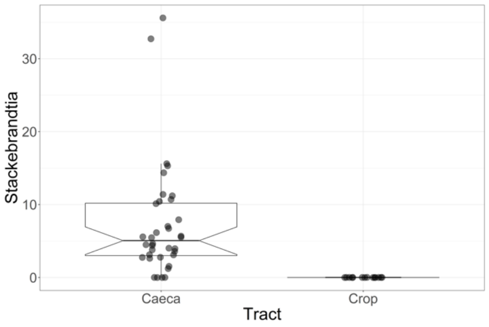

Figure S8. Boxplot of significant $\log_2$ fold changes (caeca vs crop). The panels show boxplots of statistically significant $\log_2$ fold changes in genera abundance between caeca and crop of chickens, for genera with a discordant change in shotgun and 16S samples. Shotgun samples on the left and 16S samples on the right. Points outside the box notch are statistical outliers.

Genus	Shotgun genera abundances	16S genera abundances
**Borrelia**	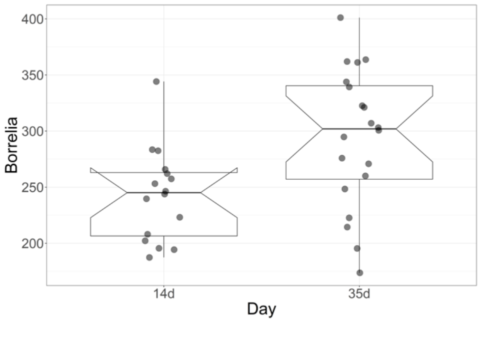	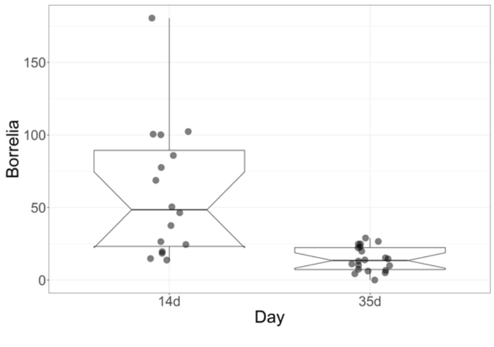

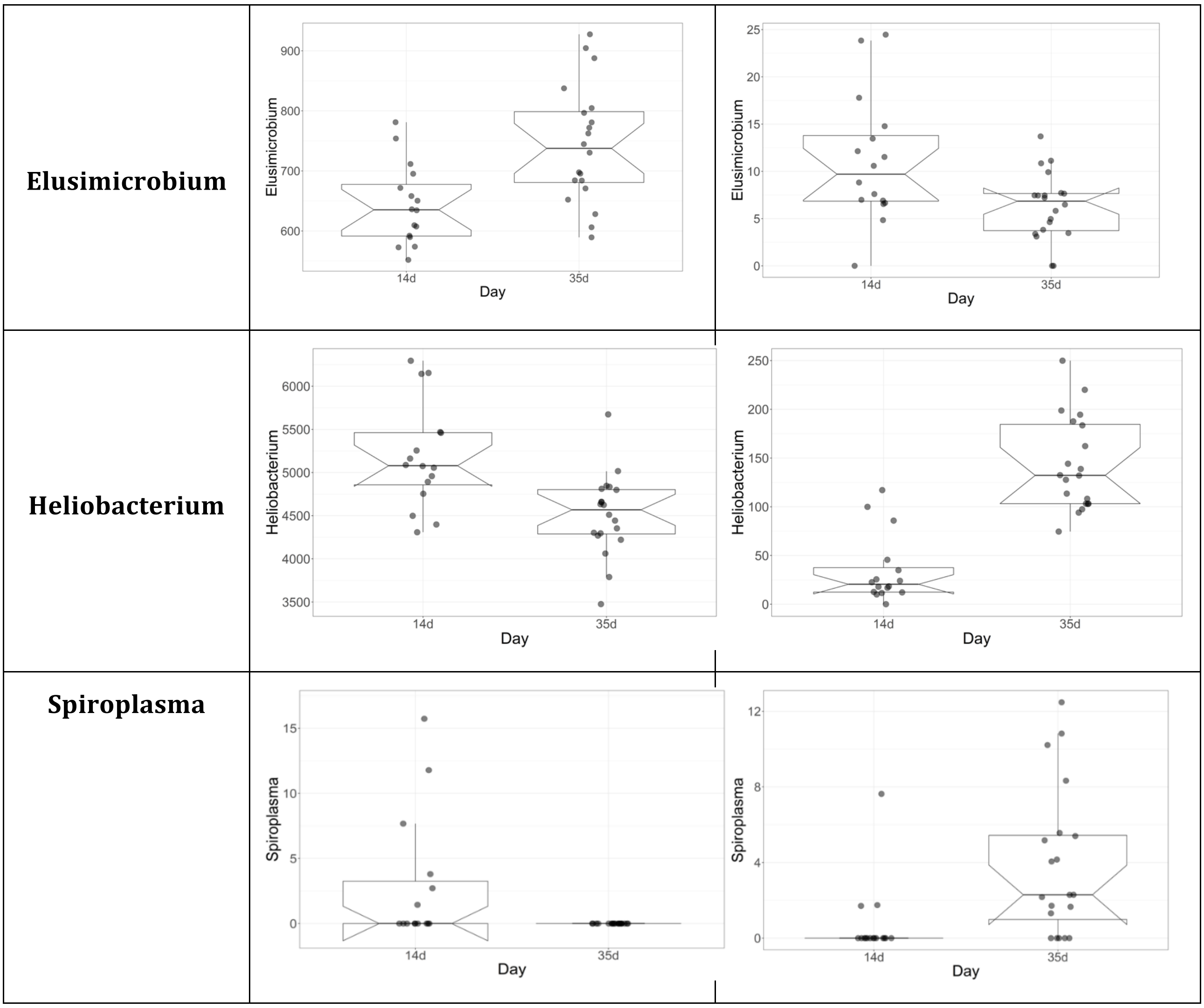

Figure S9. Boxplot of significant $\log_2$ fold changes (14th vs 35th day). The panels show boxplots of statistically significant $\log_2$ fold changes in genera abundance between 14th and 35th day, for genera with a discordant change in shotgun and 16S samples. Shotgun samples on the left and 16S samples on the right. Points outside the box notch are statistical outliers.

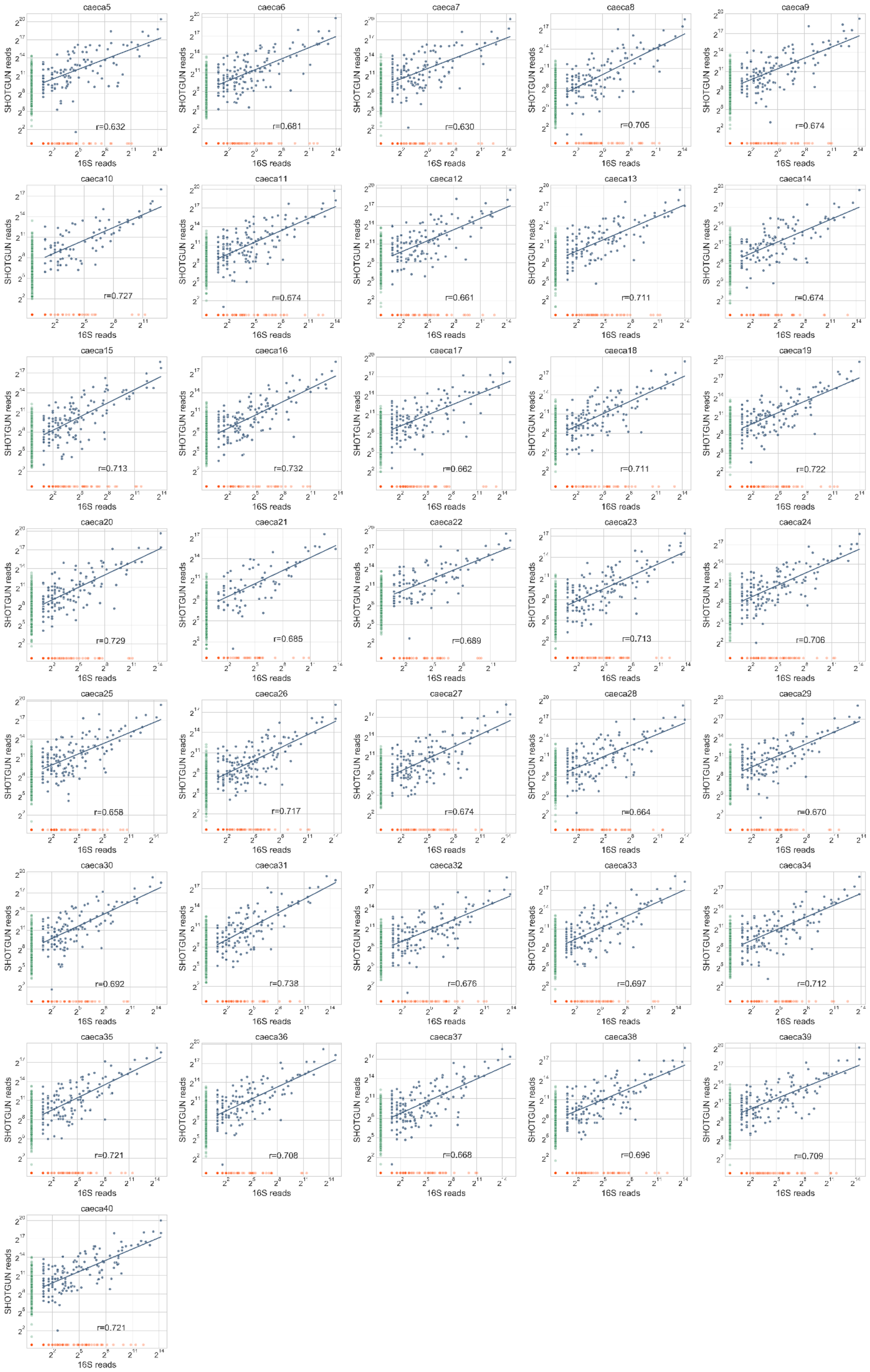
caeca5
r=0.632
caeca6
r=0.681
caeca7
r=0.630
caeca8
r=0.705
caeca9
r=0.674
caeca10
r=0.727
caeca11
r=0.674
caeca12
r=0.661
caeca13
r=0.711
caeca14
r=0.674
caeca15
r=0.713
caeca16
r=0.732
caeca17
r=0.662
caeca18
r=0.711
caeca19
r=0.722
caeca20
r=0.729
caeca21
r=0.685
caeca22
r=0.689
caeca23
r=0.713
caeca24
r=0.706
caeca25
r=0.658
caeca26
r=0.717
caeca27
r=0.674
caeca28
r=0.664
caeca29
r=0.670
caeca30
r=0.692
caeca31
r=0.738
caeca32
r=0.676
caeca33
r=0.697
caeca34
r=0.712
caeca35
r=0.721
caeca36
r=0.708
caeca37
r=0.668
caeca38
r=0.696
caeca39
r=0.709
caeca40
r=0.721
SHOTGUN reads
16S reads

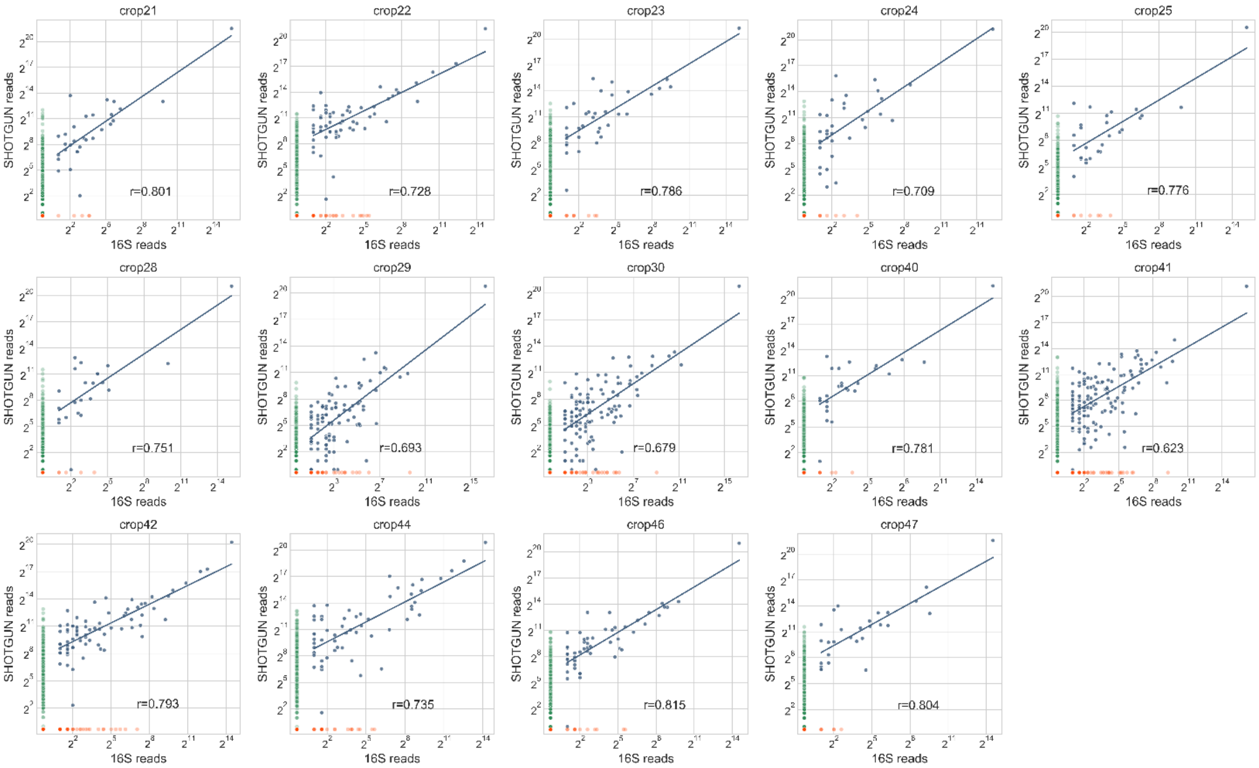

Figure S10. **Scatter plot of 16S and SHOTGUN genera abundances for all samples with more than 500000 reads.** Blue dots stand for the abundance of genera detected by both sequencing strategies in (Top) caeca and (Bottom) crop samples. The other dots refer to genera detected exclusively by only one strategy (ORANGE for 16S and GREEN for shotgun). Pearson's correlation coefficients are computed only on the common genera. Log_2 scale is adopted for both axis.

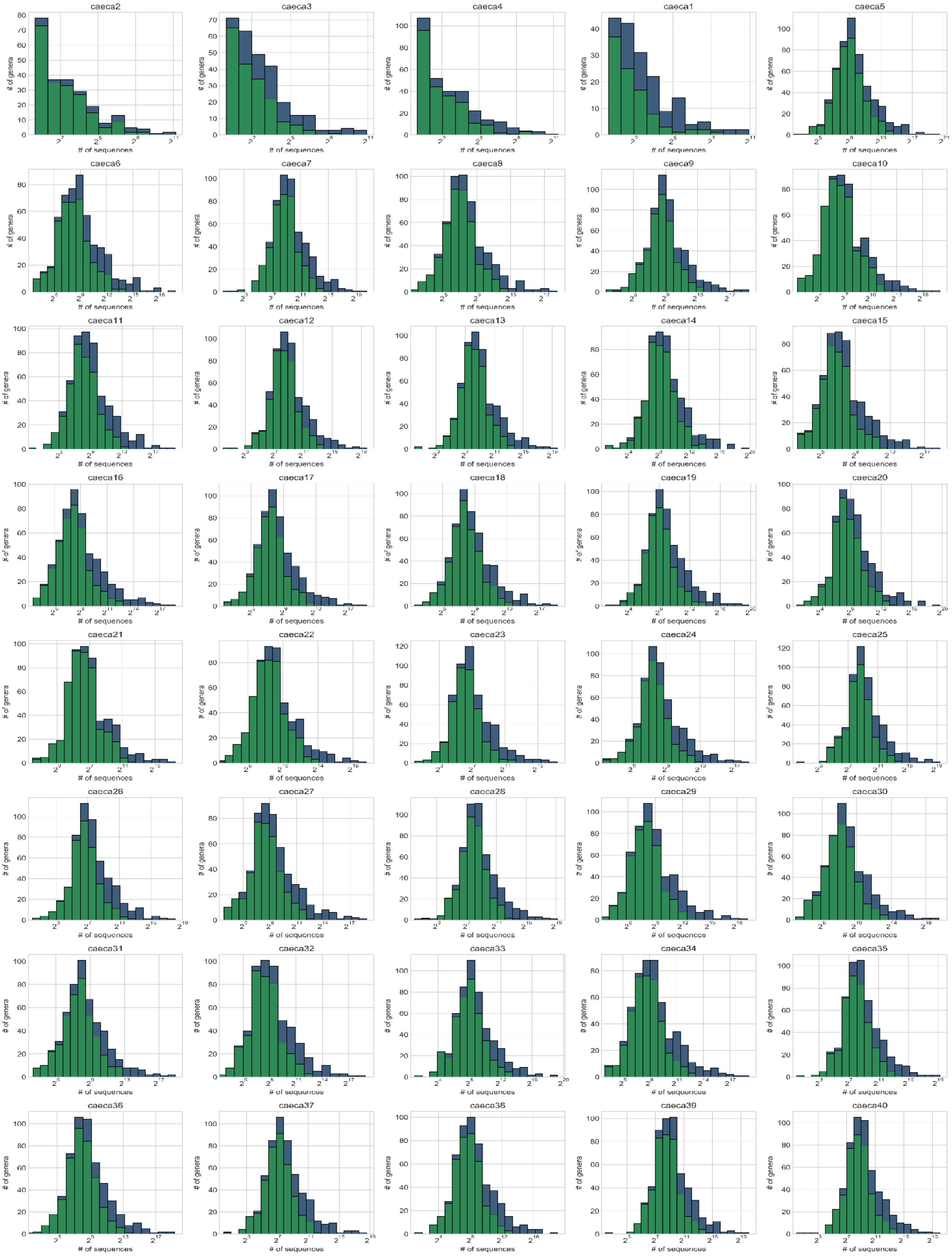

Figure S11. **Preston plot of genera abundances in all shotgun samples from caeca.** Histograms display stacked bars, where every column is divided in a part corresponding to the abundance of genera detected by both sequencing strategies (BLUE) and the other part is relative to genera detected exclusively by shotgun sequencing (GREEN). Logarithmic (log_2) scale helps to recognize that rarest genera identified by shotgun sequencing are almost not detected by 16S sequencing.

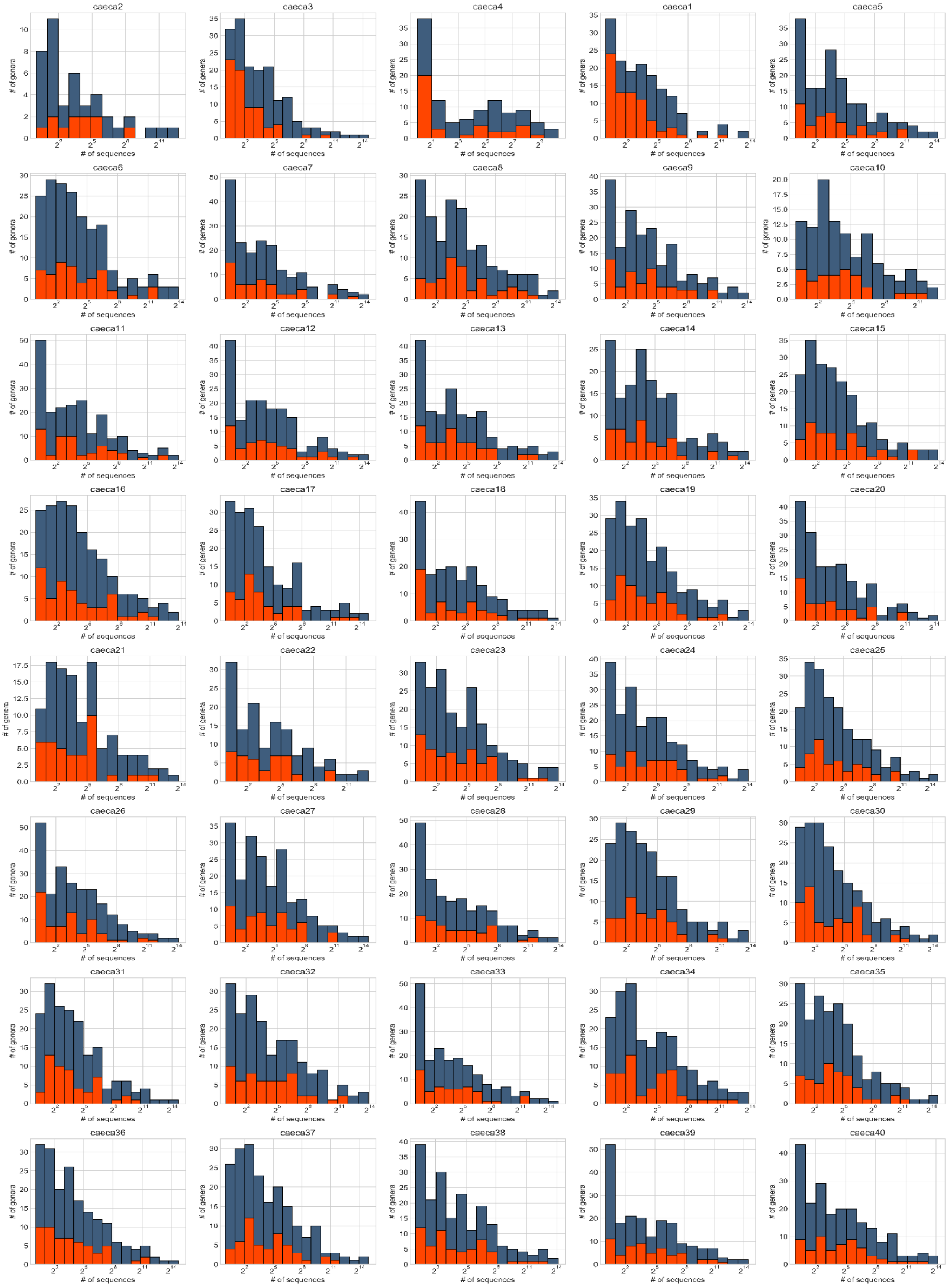

Figure S12. **Preston plot of genera abundances in all 16S samples from caeca.** Histograms display stacked bars, where every column is divided in a part corresponding to the abundance of genera detected by both sequencing strategies (BLUE) and the other part is relative to genera detected exclusively by shotgun sequencing (ORANGE).

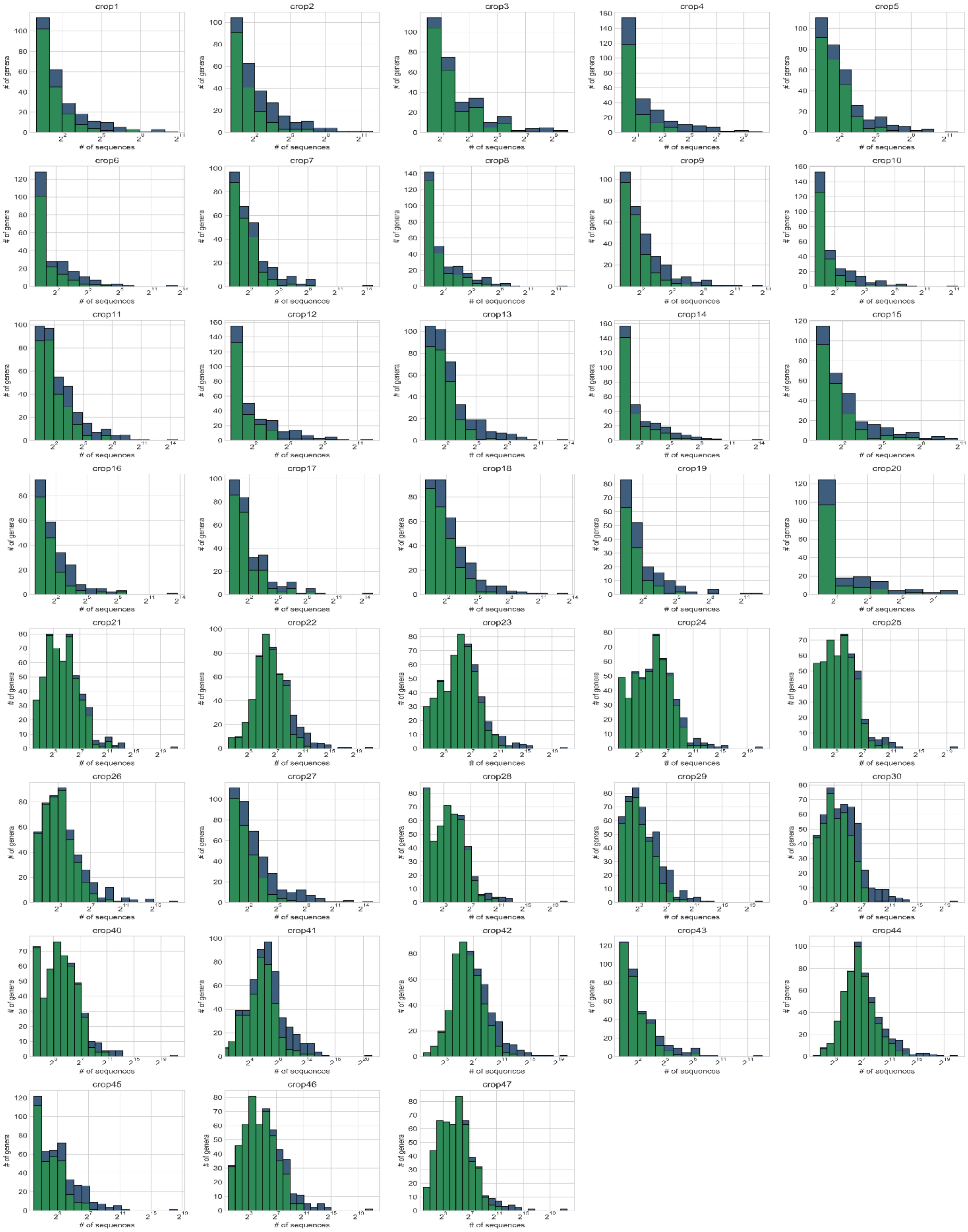

Figure S13. Preston plot of genera abundances in all shotgun samples from crop. Histograms display stacked bars, where every column is divided in a part corresponding to the abundance of genera detected by both sequencing strategies (BLUE) and the other part is relative to genera detected exclusively by shotgun sequencing (GREEN). Logarithmic (log_2) scale helps to recognize that rarest genera identified by shotgun sequencing are almost not detected by 16S sequencing.

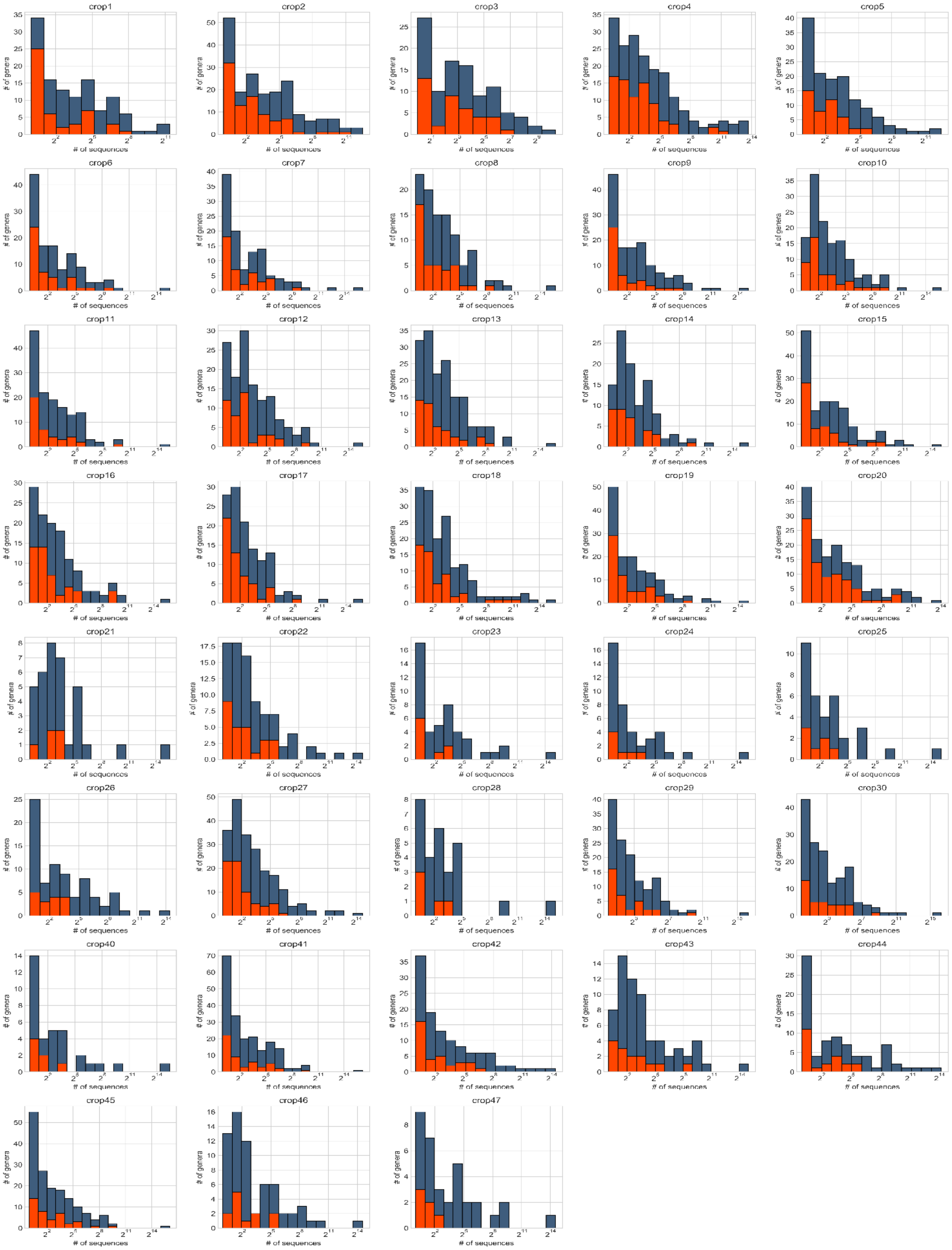

Figure S14. **Preston plot of genera abundances in all 16S samples from crop.** Histograms display stacked bars, where every column is divided in a part corresponding to the abundance of genera detected by both sequencing strategies (BLUE) and the other part is relative to genera detected exclusively by shotgun sequencing (ORANGE).

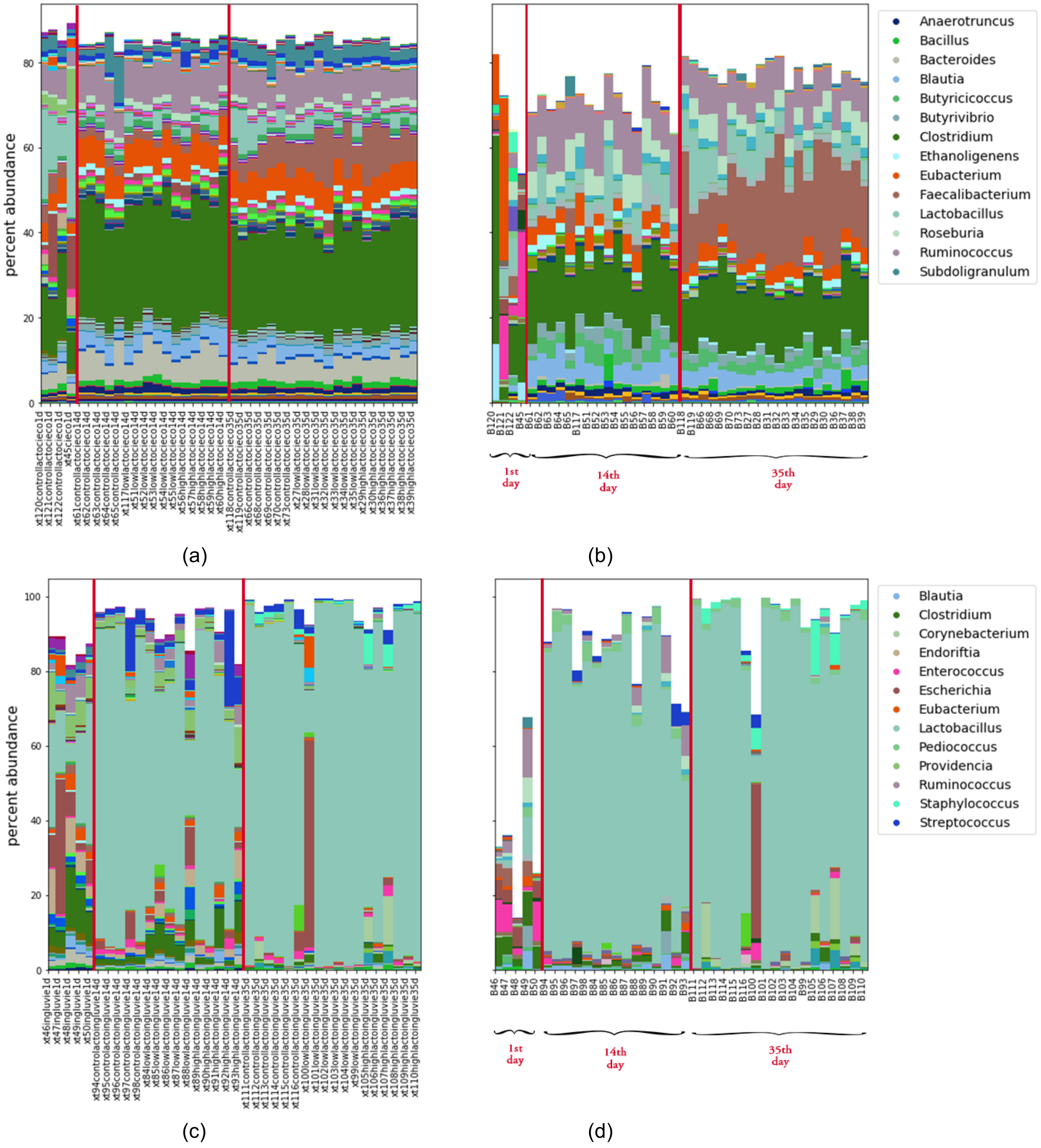

Figure S15. Proportional genera abundance of samples. Samples are ordered from 1st to 14th and 35th day on the left-right direction, for (a,b) CAECA and (c,d) CROP. Shotgun samples are in a-c, 16S samples in b-d. The height of single coloured portion of a bar represents the percentage of a genus abundance in that sample. In the legend, we labelled only those genera whose abundance exceeded 1% on average. See Table S for ID-label correspondence.

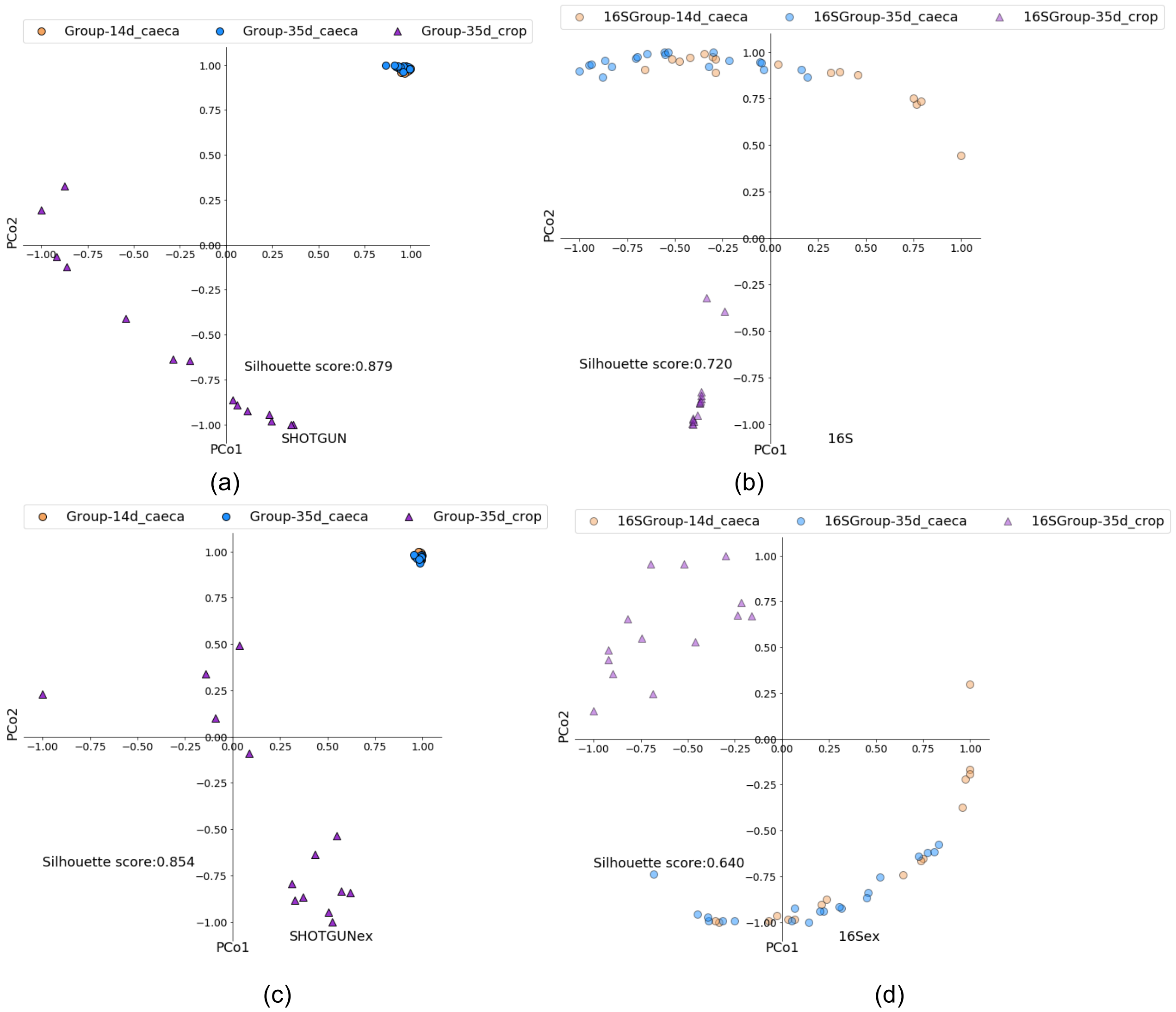

Figure S16. **PCoA of all samples (with more than 500000 reads)**. PCoA is based on the beta-diversities between samples (Bray-Curtis metric), computed on genera abundances normalized by DESnorm. Gold-Cyan is for 14th-35th day from caeca, Violet for 35th day from crop of chickens. (a) Full shotgun samples, (b) full 16S samples, (c) samples with shotgun exclusive genera, (d) samples with 16S exclusive genera.

	Shotgun # of reads (%)	16S # of reads (%)	# of genera
	**Caeca**		
**Only in shotgun**	9.0 ± 1.3	0	400 (373 – 455)
**Only in 16S**	0	12 ± 3	54 (30 – 72)
**In both**	91.0 ± 1.3	89 ± 3	133 (79 – 160)
	**Crop**		
**Only in shotgun**	2.0 ± 1.4	0	450 (371 – 490)
**Only in 16S**	0	0.6 ± 0.5	19 (5 – 51)
**In both**	98.0 ± 1.4	99.4 ± 0.6	59 (23 – 148)

Table S1. 1st-2nd column: Average and standard deviation of the percent abundances of each sample, considering the reads mapping to genera detected only by shotgun, only by 16S and by both sequencing strategies. 3rd column: Number (and range) of genera detected only by shotgun samples, only by 16S samples and those detected by both strategies.

LABEL: intestinal tract	SHOTGUN	16S	SHOTGUNex	16Sex
**SS**	0.88 ± 0.03	0.72 ± 0.02	0.85 ± 0.04	0.640 ± 0.017

Table S2. Average Silhouette Score and standard error of the mean on Bray-Curtis PCoA of genera abundances according to intestinal tract (caeca vs crop), on counts normalized by DESeq2. Datasets: full set of genera detected by shotgun (SHOTGUN) and 16S (16S), genera detected exclusively by shotgun (SHOTGUNex) and 16S (16Sex) strategies.

LABEL: sampling time	SHOTGUN	16S	SHOTGUNex	16Sex
**Mean SS**	0.51 ± 0.04	0.52 ± 0.03	0.45 ± 0.03	0.16 ± 0.05

Table S3. Average Silhouette Score and standard error of the mean on Bray-Curtis PCoA of genera abundances according to sampling time (14th vs 35th day), on counts from caeca normalized by DESeq2. Datasets: full set of genera detected by shotgun (SHOTGUN) and 16S (16S), genera detected exclusively by shotgun (SHOTGUNex) and 16S (16Sex) strategies.

Year	Study	Shotgun reads	16S reads	Better results on:
		Effectively used for taxonomic profiling	Effectively used for taxonomic profiling	
2011	Shah	~ 1000	~ 25 000	16S
2017	Tessler	~ 360 000*	~ 10 000	16S
2018	Laudadio	~ 700 000	~ 170 000	Shotgun
2018	Campanaro	~ 100 000	~ 1 500 000	Shotgun
2019	Our work	~ 1 500 000	~ 60 000	Shotgun

Table S4. Number of metagenomic reads in references. Average number of reads per sample in our current work (last line), compared to other studies in literature. All values are estimated from textual indications or from deposited data where the number of reads was not displayed explicitly in a paper. *Authors report 12M reads per sample on average. By manual inspection of deposited data, we found that only about 3% of the reads were used for most samples, thus we reported this value as a more realistic estimate of reads available for analysis.

16S metagenome label	Shotgun metagenome label	Sample label	Sampling time	Intestinal tract	16S reads	Shotgun reads
B120	xt120controllactocieco1d	2	1st day	Caeca	11705	10413
B121	xt121controllactocieco1d	3	1st day	Caeca	50639	12532
B122	xt122controllactocieco1d	4	1st day	Caeca	9001	12190
B45	xt45cieco1d	1	1st day	Caeca	40706	7654
B61	xt61controllactocieco14d	5	14th day	Caeca	91151	4724785
B62	xt62controllactocieco14d	6	14th day	Caeca	83317	3016588
B63	xt63controllactocieco14d	7	14th day	Caeca	94597	4754691
B64	xt64controllactocieco14d	8	14th day	Caeca	86520	1438466
B65	xt65controllactocieco14d	9	14th day	Caeca	85242	2914290
B56	xt56highlactocieco14d	16	14th day	Caeca	69719	1645975
B57	xt57highlactocieco14d	17	14th day	Caeca	120954	1942394
B58	xt58highlactocieco14d	18	14th day	Caeca	82806	2197678
B59	xt59highlactocieco14d	19	14th day	Caeca	76405	3234991
B60	xt60highlactocieco14d	20	14th day	Caeca	81386	2331381
B117	xt117lowlactocieco14d	10	14th day	Caeca	35330	806601
B51	xt51lowlactocieco14d	11	14th day	Caeca	87713	3033885
B52	xt52lowlactocieco14d	12	14th day	Caeca	86532	3014934
B53	xt53lowlactocieco14d	13	14th day	Caeca	79941	3115551
B54	xt54lowlactocieco14d	14	14th day	Caeca	78251	2827872
B55	xt55lowlactocieco14d	15	14th day	Caeca	78678	1522063
B118	xt118controllactocieco35d	21	35th day	Caeca	41226	714039
B119	xt119controllactocieco35d	22	35th day	Caeca	39615	3147868
B66	xt66controllactocieco35d	23	35th day	Caeca	92539	725666
B68	xt68controllactocieco35d	24	35th day	Caeca	92141	1725076
B69	xt69controllactocieco35d	25	35th day	Caeca	97479	2972671
B70	xt70controllactocieco35d	26	35th day	Caeca	83045	1045439
B73	xt73controllactocieco35d	27	35th day	Caeca	90889	2068229
B29	xt29highlactocieco35d	35	35th day	Caeca	82398	2633233
B30	xt30highlactocieco35d	36	35th day	Caeca	76510	2460932
B36	xt36highlactocieco35d	37	35th day	Caeca	95995	1523653
B37	xt37highlactocieco35d	38	35th day	Caeca	91060	1992262
B38	xt38highlactocieco35d	39	35th day	Caeca	100136	3720998
B39	xt39highlactocieco35d	40	35th day	Caeca	103787	3930570
B27	xt27lowlactocieco35d	28	35th day	Caeca	77498	2195624
B28	xt28lowlactocieco35d	29	35th day	Caeca	68610	2252007
B31	xt31lowlactocieco35d	30	35th day	Caeca	75796	3044478
B32	xt32lowlactocieco35d	31	35th day	Caeca	70171	2243584
B33	xt33lowlactocieco35d	32	35th day	Caeca	71281	1665479
B34	xt34lowlactocieco35d	33	35th day	Caeca	99075	2442079
B35	xt35lowlactocieco35d	34	35th day	Caeca	85868	2046583
B46	xt46ingluvie1d	1	1st day	Crop	11624	6629
B47	xt47ingluvie1d	2	1st day	Crop	33095	10735
B48	xt48ingluvie1d	3	1st day	Crop	5333	5950

B49	xt49ingluvie1d	4	1st day	Crop	73617	5410
B50	xt50ingluvie1d	5	1st day	Crop	15365	9866
B94	xt94controlactoingluvie14d	6	14th day	Crop	49805	13580
B95	xt95controlactoingluvie14d	7	14th day	Crop	77085	29151
B96	xt96controlactoingluvie14d	8	14th day	Crop	60144	43574
B97	xt97controlactoingluvie14d	9	14th day	Crop	44876	23431
B98	xt98controlactoingluvie14d	10	14th day	Crop	64429	25127
B89	xt89highlactoingluvie14d	16	14th day	Crop	65564	16640
B90	xt90highlactoingluvie14d	17	14th day	Crop	66256	32795
B91	xt91highlactoingluvie14d	18	14th day	Crop	95142	20752
B92	xt92highlactoingluvie14d	19	14th day	Crop	46368	11398
B93	xt93highlactoingluvie14d	20	14th day	Crop	37553	2225
B84	xt84lowlactoingluvie14d	11	14th day	Crop	47359	39240
B85	xt85lowlactoingluvie14d	12	14th day	Crop	57225	10836
B86	xt86lowlactoingluvie14d	13	14th day	Crop	74967	23982
B87	xt87lowlactoingluvie14d	14	14th day	Crop	63427	27418
B88	xt88lowlactoingluvie14d	15	14th day	Crop	52399	8660
B111	xt111controllactoingluvie35d	21	35th day	Crop	49592	3151076
B112	xt112controllactoingluvie35d	22	35th day	Crop	35581	3249035
B113	xt113controllactoingluvie35d	23	35th day	Crop	37015	2774971
B114	xt114controllactoingluvie35d	24	35th day	Crop	42153	2725740
B115	xt115controllactoingluvie35d	25	35th day	Crop	38620	1526674
B116	xt116controllactoingluvie35d	26	35th day	Crop	27807	301329
B105	xt105highlactingluvie35d	42	35th day	Crop	38680	1932019
B106	xt106highlactingluvie35d	43	35th day	Crop	36865	57146
B107	xt107highlactingluvie35d	44	35th day	Crop	34767	3433364
B108	xt108highlactingluvie35d	45	35th day	Crop	72803	360581
B109	xt109highlactingluvie35d	46	35th day	Crop	27229	2295326
B110	xt110highlactingluvie35d	47	35th day	Crop	25633	3385569
B100	xt100lowlactingluvie35d	27	35th day	Crop	44808	54756
B101	xt101lowlactoingluvie35d	28	35th day	Crop	38024	2316485
B102	xt102lowlactoingluvie35d	29	35th day	Crop	84585	1842157
B103	xt103lowlactoingluvie35d	30	35th day	Crop	88162	1912431
B104	xt104lowlactoingluvie35d	40	35th day	Crop	44788	2955926
B99	xt99lowlactoingluvie35d	41	35th day	Crop	78598	2660118

Table S5. 16S and shotgun metagenome labels of the samples tested and sample labels with indication of sampling times, target intestinal tract and treatment. Number of reads is the value computed by MG-RAST platform (reads mapping to Bacteria domain). Red numbers of reads are lower than 500000, indicating samples excluded from the comparative analyses, as indicated in the main text.
